# Compounds Isolated from *Wikstroemia taiwanensis* Regulate Bone Remodeling by Modulating Osteoblast and Osteoclast Activities

**DOI:** 10.3389/fphar.2021.670254

**Published:** 2021-07-13

**Authors:** Zuha Imtiyaz, Yi-Tzu Lin, Fang-Yu Liang, Wen-Fei Chiou, Mei-Hsien Lee

**Affiliations:** ^1^ PhD in Clinical Drug Development of Herbal Medicine, College of Pharmacy, Taipei Medical University, Taipei, Taiwan; ^2^ Department of Pathology, School of Medicine, University of Maryland, Baltimore, Baltimore, MD, United States; ^3^ Institute of Biological Chemistry, Academia Sinica, Taipei, Taiwan; ^4^ Graduate Institute of Pharmacognosy, College of Pharmacy, Taipei Medical University, Taipei, Taiwan; ^5^ National Research Institute of Chinese Medicine, Ministry of Health and Welfare, Taipei, Taiwan; ^6^ Center for Reproductive Medicine & Sciences, Taipei Medical University Hospital, Taipei, Taiwan

**Keywords:** bone remodeling, osteoblasts, osteoclasts, *Wikstroemia taiwanensis*, astragalin, kaempferol 3-*O*-*β*-d-apiofuranosyl-(1→6)-*β*-d-glucopyranoside

## Abstract

Bone remodeling, a dynamic process in which bone formation by osteoblast is preceded by bone resorption by osteoclast, is a vital physiological process for maintaining bone mass and strength, imbalances in which could precipitate osteoporosis. Due to the unilateral mechanism of the existing bone remodeling drugs, identifying compounds that could regulate the balance between osteoclast and osteoblast could improve the treatment of osteoporosis. Here, we show that compounds isolated from *Wikstroemia taiwanensis* modulate osteoclast and osteoblast activities. Specifically, astragalin (**1**) and kaempferol 3-*O*-*β*-D-apiofuranosyl-(1→6)-*β*-D-glucopyranoside (**2**), besides increasing mineral deposition, increased alkaline phosphatase activity (137.2% for **1** and 115.8% for **2**) and ESR-α expression (112.8% for **1** and 122.5% for **2**) in primary human osteoblasts. In contrast, compounds **1**, **2**, **3**, and **5** inhibited tartrate-resistant acid phosphatase (TRAP) activity in receptor activator of nuclear factor-κB ligand-induced osteoclasts by 40.8, 17.1, 25.9, and 14.5% and also decreased the number of TRAP-positive cells by 51.6, 26.8, 20.5, and 18.6%, respectively. Our findings, therefore, showed that compounds isolated from *W. taiwanensis* could increase osteoblast activity while simultaneously decreasing osteoclast activity, and hence, warrant further evaluation for development as anti-osteoporosis agents.

## Introduction

Osteogenesis or bone remodeling is a physiological process in which bone tissue undergoes continuous metabolism. It involves the removal of old and damaged mineralized bone by osteoclasts and the formation of new bone by osteoblasts on the matrix, which further becomes mineralized in order to maintain the overall integrity and stability of the bone (Eriksen, 2010). Osteoblast and osteoclast cells function in small anatomic units called basic multicellular units, where bone remodeling such as resorption followed by mineralization takes place. Osteoclastogenesis, the formation of the osteoclast bone-resorbing cells, is regulated by two major factors, the receptor of nuclear factor-κB (RANK) ligand (RANKL), and osteoprotegerin (OPG). RANKL is produced by osteoblast and binds to the RANK present on pre-osteoclasts leading to the formation of a fused polykaryon, a multinuclear cell that further matures into functional and mature osteoclasts (Crockett et al., 2011). In contrast, OPG, which is similarly produced by osteoblasts, is a decoy receptor of RANKL and, therefore, can inhibit osteoclastogenesis (Chen et al., 2018). The inhibition caused by OPG can directly affect osteoclast formation, differentiation, activation, and survival. Hence, the appropriate RANKL/OPG ratio is essential for balanced bone remodeling, and since they are both produced by osteoblasts, it implies that osteoblasts have a key role in maintaining balanced bone remodeling (Owen and Reilly, 2018). Given the exquisitely regulated nature of bone remodeling, imbalance in this physiological process could lead to the onset of conditions such as Buchem disease and osteoporosis (Niedzwiedzki and Filipowska, 2015).

Osteoporosis is a disease of bones that is characterized by reduced bone mass and weak bone microarchitecture, making them susceptible to fractures. It is a critical public health threat affecting approximately 200 million people worldwide, the majority of whom are postmenopausal women. Although several treatment strategies are currently available to combat osteoporosis, they are fraught by their unilateral mechanism-of-action (they modulate either osteoblast or osteoclast activity, but very seldom both). Given the importance of the balance between osteoclast and osteoblast activity in maintaining the proper bone function, identifying therapeutic strategies that could increase bone formation while concomitantly decreasing bone resorption could improve the management of osteoporosis.

Plants are an essential source of drug discovery, and plants derived from the *Wikstroemia* genus are known to possess phytochemicals with numerous medicinal benefits. *Wikstroemia dolichantha* Diels (Thymelaeaceae), which contains the biflavonoid, chamaejasmine, was found to be effective against atopic dermatitis in SKH-1 hairless mice (Kim et al., 2019). *W. chamaedaphne* Meisn. has been used to treat cough, edema, schizophrenia, hepatitis, and antifertility in traditional Chinese medicines (Qian et al., 2020). *W. indica* (L.) C.A. Mey. has been used as folk medicine to treat arthritis and bronchitis in China for a very long time (Li et al., 2009). Besides, compounds isolated from the *Wikstroemia* genus of plants exhibited antiviral activities against the hepatitis B virus and hepatitis C virus (Khong et al., 2012; Li et al., 2018). They also protected PC12 neuronal cells against oxygen and glucose deprivation/restoration-induced injury (Yao et al., 2017; Yao et al., 2019). These studies provided compelling evidence for the medicinal properties of *Wikstroemia* genus plants. *Wikstroemia taiwanensis* C.E. Chang (Thymelaeaceae) (WT), a plant endemic to Taiwan, is a shrub found in the Pingtung area of southern Taiwan. A study showed that bioflavonoids such as wikstaiwanones A–C, sikokianins B and C, isochamaejasmin and methyl 4-hydroxybenzoate were isolated from the EtOAc-soluble fraction. Sikokianin B and C exhibited antitubercular activity against *Mycobacterium tuberculosis* (Chen et al., 2012). WT was identified through our screening to possess osteogenic potential (Supplementary Table S1). However, whether WT enhances bone formation is unknown. Given the need to identify compounds that simultaneously modulate both osteoblast and osteoclast activities for the treatment of osteoporosis, we examined the effect of WT osteoblasts activity. Our results show that WT increases osteoblast activity while at the same time, decrease osteoclast activity, making it an ideal candidate for the treatment of osteoporosis.

## Materials and Methods

### Reagents and General Instrumentation

Alizarin red S, ascorbic acid, bovine serum albumin, dexamethasone, 3-(4,5-dimethylthiazol-2-yl)-2,5-diphenyltetrazolium bromide (MTT), 17β-estradiol, β-glycerophosphate (β-GP), sodium carbonate, sodium hydroxide, and trypan blue were purchased from Merck (Darmstadt, Germany). ACS/liquid chromatographic (LC)-grade acetone and methanol were purchased from Echo Chemical (Miaoli, Taiwan). Cetylpyridinium chloride, *p*-nitrophenyl phosphate, paraformaldehyde, sodium bicarbonate, and sodium phosphate were purchased from Mallinckrodt Pharmaceuticals (St. Louis, MO, United States). Dimethyl sulfoxide (DMSO), Dulbecco’s modified Eagle’s medium (DMEM), Dulbecco's phosphate-buffered saline (PBS), fetal bovine serum (FBS), penicillin-streptomycin, Triton X-100, and trypsin were purchased from Thermo Fisher Scientific (Waltham, MA United States). Recombinant mouse TRANCE/RANKL/TNFSF11 was purchased from R&D Systems (Minneapolis, MN, United States). Sodium acetate and sodium tartrate were purchased from Tokyo Chemical Industry (TCI, Tokyo, Japan). The Synergy™ HT multi-detection microplate reader was purchased from BioTek (Winooski, VT, United States). The high-performance LC (HPLC) system (consisting of an L-7100 pump and L-7455 diode array detector), and a UV-2800 UV-VIS spectrophotometer was purchased from Hitachi (Tokyo, Japan). The Orbitrap Elite Mass Spectrometer was purchased from Thermo Fisher Scientific (Waltham, United States). Nuclear magnetic resonance (NMR) instruments of 300 and 500 MHz were purchased from Bruker (Karlsruhe, Germany).

### Plant Extract Preparation

Leaves of WT were collected from Mutan, Pingtung County, Taiwan and identified by Ih-Sheng Chen, School of Pharmacy, Kaohsiung Medical University (Kaohsiung, Taiwan). An herbarium voucher specimen (M-393) was deposited in the Graduate Institute of Pharmacognosy, Taipei Medical University (Taipei, Taiwan). WT leaves (WTL, 1,280 g) were extracted with 70% acetone three times for seven days each time. After rotary evaporation and vacuum drying, the WTL extract (250 g) was obtained.

### Compound Isolation

The WTL extract (250 g) was suspended in an aqueous solution and fractionated through a Diaion^®^ HP-20 column (250–850 μm, Merck) to obtain six fractions, WTL 1-1∼1-6. After bio-guided fractionation and isolation, the active WTL 1-4 fraction was subjected to a Cosmosil^®^ C-18 column (75 μm, Nacalai Tesque, Kyoto, Japan) eluted with an H_2_O-100% methanol (MeOH) gradient, to yield 13 sub-fractions (WTL 1-3-1∼1-3-13). Sub-fractions WTL 1-4-10 and 1-4-8 were further purified using a Cosmosil^®^ C-18 column and a semipreparative C-18 reverse-phase HPLC column (Biotic Aqu-ODS-W 5 μm, 10 × 250 mm; Biotic Chemical, New Taipei City, Taiwan) with 50% MeOH as the mobile phase to isolate **1** (51.1 mg) and **2** (38.0 mg). The active WTL 1-2 fraction was subjected to a Sephadex^®^ LH-20 column (18–111 μm, Merck KGaA) eluted with an H_2_O-100% MeOH gradient, to yield 11 sub-fractions (WTL 1-2-1∼1-2-11). Sub-fraction WTL 1-2-4 was further separated using a semipreparative C-18 reverse-phase HPLC column with 25% MeOH as the mobile phase to isolate **3** (6.6 mg) and **4** (13.0 mg). The active WTL 1-3 fraction was subjected to a Cosmosil^®^ C-18 column eluted with an H_2_O-100% MeOH gradient, to yield 15 sub-fractions (WTL 1-3-1∼1-3-15). Sub-fraction WTL 1-3-4 was further separated using an MCI GEL^®^ CHP20P column (75–150 μm, Merck KGaA) and semipreparative Luna PFP (2) (5 μm, 10 × 250 mm, Phenomenex, Torrance, CA) reverse-phase HPLC column with 35% MeOH as the mobile phase to isolate **5** (6.6 mg).

### Acid Hydrolysis of the Isolated Compounds

The isolated compound (1.0 mg) was hydrolyzed with hydrochloric acid into monosaccharide and polysaccharide hydrolysates. They were then separated using a Dionex^®^ high-performance anion-exchange chromatographic system (Thermo Fisher Scientific) with a Dionex™ Carbopac™ anion-exchange column (PA-10, 4.6 × 250 mm, Thermo Fisher Scientific). The mobile phase was 18 mM sodium hydroxide. Monosaccharides were identified by comparing the retention times with standards, and quantification was performed using calibration curves (Hsu et al., 2006).

### Cell Culture

Primary human osteoblasts (HOb cells, Cell Applications, San Diego, CA, United States ) were maintained in an osteoblast growth medium kit (Cell Applications) in an atmosphere of 5% CO_2_ at 37°C. In order to obtain fully differentiated HOb cells, cells were incubated with an osteoblast differentiation medium (ODM) kit from Cell Applications, containing ascorbic acid, dexamethasone, and β-GP for the mineralization assay (Mao et al., 2014).

RAW264.7 cells (American Type Culture Collection, Manassas, VA, United States) were maintained in DMEM supplemented with 10% FBS, 100 U/mL streptomycin, and 100 U/mL penicillin in an atmosphere of 5% CO_2_ at 37°C. RAW264.7 cells were induced by RANKL (50 ng/ml) for five days to differentiate into RANKL-induced osteoclasts (Yan et al., 2019). For all the cell experiments, samples were dissolved in DMSO to make the stock solutions.

### Cell Viability Analysis

HOb cells were treated with samples at various indicated concentrations for 5 days. RANKL-induced osteoclasts were treated with samples for five days. Cell viability was measured with an MTT assay performed at an absorbance of 600 nm (Mao et al., 2014).

### Alkaline Phosphatase Activity

HOb cells were treated with samples at various indicated concentrations for 5 days. Cells were washed with PBS twice and sonicated in lysis buffer containing 0.1% Triton X-100. ALP activity was measured with *p*-nitrophenyl phosphate (dissolved in 6 mM sodium bicarbonate-sodium carbonate buffer, pH 10.0) and normalized to the protein concentration. The protein concentration was measured with a bicinchoninic acid (BCA) protein assay kit (Thermo Fisher Scientific). ALP activity was assessed at an absorbance of 405 and 562 nm (Mao et al., 2014).

### Alizarin Red S Staining

HOb cells were treated with samples at various indicated concentrations for 11 days. HOb cells were washed with PBS and fixed with 4% paraformaldehyde for 20 min. Cells were washed with PBS, and then exposed to an alizarin red S solution for 20 min at room temperature. Mineralization-positive cells stained red. After taking photos, 10% cetylpyridinium chloride (dissolved in 10 mM sodium phosphate, pH 7.0) was used to quantify mineralization staining at an absorbance of 550 nm (Imtiyaz et al., 2019).

### RNA Isolation and Real-Time Quantitative Polymerase Chain Reaction

Total RNA from HOb cells was isolated using a high pure RNA isolation kit (Roche Life Science, Mannheim, Germany). An Applied Biosystems™ high-capacity complementary (c)DNA reverse-transcription kit (Thermo Fisher Scientific) was used to synthesize cDNA. A Roche™ universal probe library (UPL) probe (Roche Life Science) and SensiFAST^™^ probe no-ROX kit (Bioline, London, United Kingdom) were used for PCR amplification with the LightCycler^®^ 480 system (Roche Life Science). The primer sequences for the PCR were as follows: Runx2, forward, 5′-CAG​TGA​CAC​CAT​GTC​AGC​AA-3′ and reverse, 5′-GCT​CAC​GTC​GCT​CAT​TTT​G-3′; osterix (OSX), forward, 5′-CAT​CTG​CCT​GGC​TCC​TTG-3′ and reverse, 5′-CAG​GGG​ACT​GGA​GCC​ATA-3′; osteopontin (OPN), forward, 5′-GGG​CTT​GGT​TGT​CAG​CAG-3′ and reverse, 5′- TGC​AAT​TCT​CAT​GGT​AGT​GAG​TTT-3′; bone morphogenetic protein (BMP)-2, forward, 5′-CGG​ACT​GCG​GTC​TCC​TAA-3′ and reverse, 5′- GGA​AGC​AGC​AAC​GCT​AGA​AG-3′, bone sialoprotein (BSP), forward, 5′-GAT​TTC​CAG​TTC​AGG​GCA​GT-3′ and reverse, 5′-TCT​CCT​TCA​TTT​GAA​GTC​TCC​TCT-3′; type I collagen (Col-1), forward, 5′-AGG​TCC​CCC​TGG​AAA​GAA-3′ and reverse, 5′-AAT​CCT​CGA​GCA​CCC​TGA-3′; and GAPDH, forward, 5′-AGC​CAC​ATC​GCT​CAG​ACA​C-3′ and reverse, 5′-GCC​CAA​TAC​GAC​CAA​ATC​C-3′′. Gene expression results were analyzed by the 2^(−ΔΔCt)^ method and the Livak formula, and were normalized against GAPDH expression and expressed as relative expression compared to that of the controls (Imtiyaz et al., 2019).

### Estrogen Receptor Expression

According to the ESR (Human) α and β enzyme-linked immunosorbent assay (ELISA) Kit (Abnova, Taipei, Taiwan) manufacturer’s instructions, HOb cells were fixed and quenched, and wells were blocked. Primary antibodies (anti-ESR-α, anti-ESR-β, and anti-GAPDH) specific for target antigens were added and allowed to bind to their respective epitopes. Horseradish peroxidase (HRP)-conjugated secondary antibodies (anti-rabbit and anti-mouse immunoglobulin G) specific for the primary antibody were added and allowed to bind to their respective epitopes. The 3,3′,5,5′-tetramethylbenzidine (TMB) substrate was converted to the blue TMB diimine *via* the HRP enzyme. Upon addition of acid, the reaction was terminated, and the absorbance was read at 450 nm (Imtiyaz et al., 2019).

### Tartrate-Resistant Acid Phosphatase Activity

TRAP was evaluated as a specific biochemical marker of RANKL-induced osteoclasts (Blumer et al., 2012). RANKL-induced osteoclasts were treated with samples for five days. Cells were washed twice with PBS. TRAP activity was measured with *p*-nitrophenyl phosphate (dissolved in 40 mM sodium acetate-10 mM sodium tartrate buffer, pH 5.0). Cells were incubated for 30 min at room temperature, and then 0.1 N sodium hydroxide was added to stop the reactions. TRAP activity was determined at an absorbance of 410 nm (Yan et al., 2019).

### Tartrate-Resistant Acid Phosphatase Staining

Osteoclastogenesis was confirmed by TRAP staining with a leukocyte acid phosphatase kit from Sigma (St. Louis, MO, United States). According to the manufacturer's instructions, the TRAP enzyme from RANKL-induced osteoclasts cleaves the substrate, forming a red azoic dye with a purplish-red color that can be detected with an Eclipse TS100 inverted microscope (Nikon Instruments, Tokyo, Japan). Mature osteoclasts were identified as TRAP-positive multinucleated cells (MNCs) containing five or more nuclei at the end of the culture period (Yan et al., 2019).

### Statistical Analysis

For all parameters measured, values for all samples in different experimental conditions were averaged, and the standard deviation (SD) was calculated. Statistical significance of differences between groups was determined with a one-way analysis of variance (ANOVA) followed by a post-hoc Student-Newman-Keuls method used to determine differences among multiple pairs.

## Results

### Effects of Various Parts of *Wikstroemia taiwanensis* and Fractions from the Crude Extract on Cell Viability and Alkaline Phosphatase Activity

**FIGURE 1:**
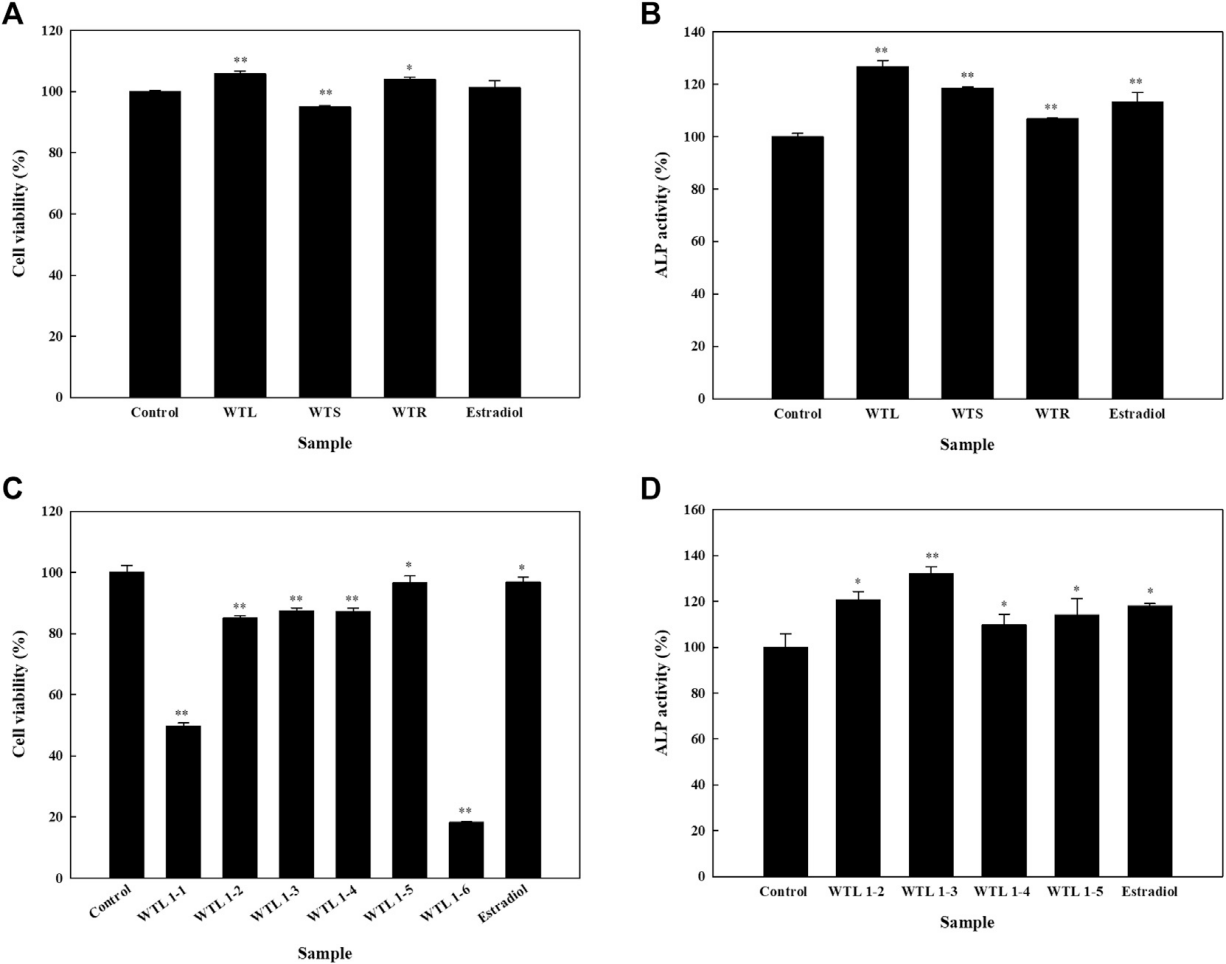
Effects of crude extracts of various parts of *Wikstroemia taiwanensis* and fractions from the active extract on cell viability and alkaline phosphatase (ALP) activity of human osteoblast cells. Cells were seeded in 96-well plates, and after 24 h, samples (100 μg/ml) were added. **(A)** Cell viability and **(B)** ALP activity assays were performed using leaves (WTL), stems (WTS), and roots of *W. taiwanensis* (WTR). **(C)** Cell viability and **(D)** ALP activity after treating cells with the WTL fraction for five days. Experiments were carried out in triplicate. * *p* < 0.05 and ** *p* < 0.01; data are presented as the mean ± standard deviation.

In order to select the part of the plant for extraction, leaves (WTL), stems (WTS), and roots (WTR) from the plant were extracted in small batches using 70% acetone. None of these three extracts caused the cell viability to decrease to below the 80% threshold (Figure 1A); hence ALP activity was analyzed, and results showed that WTL, WTS, and WTR respectively increased ALP activity up to 126.7, 118.5, and 106.9% (Figure 1B). These results indicate that WTL showed the highest activity among all three parts, and thus we used the WTL extract extraction and compound isolation. The WTL extract was obtained using 70% acetone as the extractant. Compound isolation was performed by a bio-guided method. Thus the extract was subjected to a Diaion^®^ HP-20 column, and six fractions (WTL 1-1∼1-6). The cytotoxic effects of these fractions were analyzed, and WTL 1-2∼1-5 did not affect cell viability beyond the threshold (Figure 1C). Next, we analyze the effect of the fractions on ALP activity. All four fractions significantly increased ALP activity, with WTL 1-3 showing the highest increase (Figure 1D).

### Isolated Compounds from *Wikstroemia taiwanensis* Leaves

**FIGURE 2:**
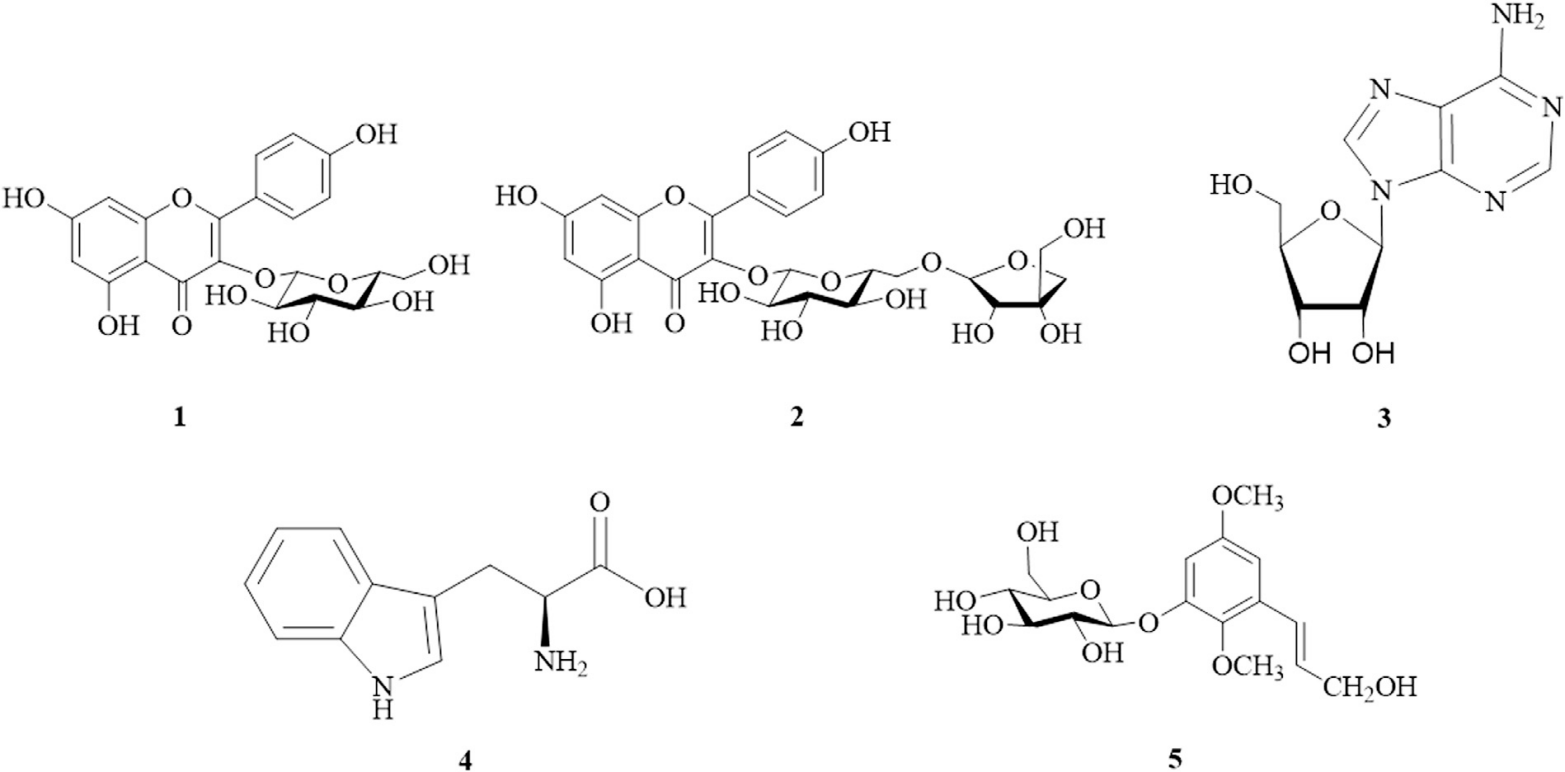
Structures of isolated compounds from *Wikstroemia taiwanensis.* **1**, astragalin; **2**, kaempferol 3-*O*-*β*-d-apiofuranosyl-(1→6)-*β*-d-glucopyranoside; **3**, adenosine; **4**, tryptophan; and **5**, 2,5-dimethoxy-3-*O*-*β*-d-glucopyranosyl cinnamic alcohol.

Column chromatography and semipreparative HPLC were used for bio-guided fractionation, isolation, and purification of compounds from the WTL 1-2, 1-3, and 1-4 fractions (Supplementary Chart S1). On the basis of physical and spectroscopic techniques, we elucidated five major compounds and compared their 1D and 2D NMR data with the literature (Supplementary Method S1, Figures S1–S5). As shown in Supplementary Figure S1F, glucose was the dominant sugar component of compound **1** when compared to standards by an acid hydrolysis method. The compounds were identified as astragalin (**1**) (Foo et al., 2000; Deng et al., 2009), kaempferol 3-*O*-*β*-d-apiofuranosyl-(1→6)-*β*-d-glucopyranoside (**2**) (Wu et al., 2007), adenosine (**3**) (Moyroud and Strazewski, 1999), tryptophan (**4**) (Lee and Phillips, 1992), and 2,5-dimethoxy-3-*O*-*β*-d-glucopyranosyl cinnamic alcohol (**5**) (Wu et al., 2014) (Figure 2). These five isolated compounds are reported herein for the first time from WT.

### Effects of the Isolated Compounds on the Cell Viability of HOb Cells

**FIGURE 3:**
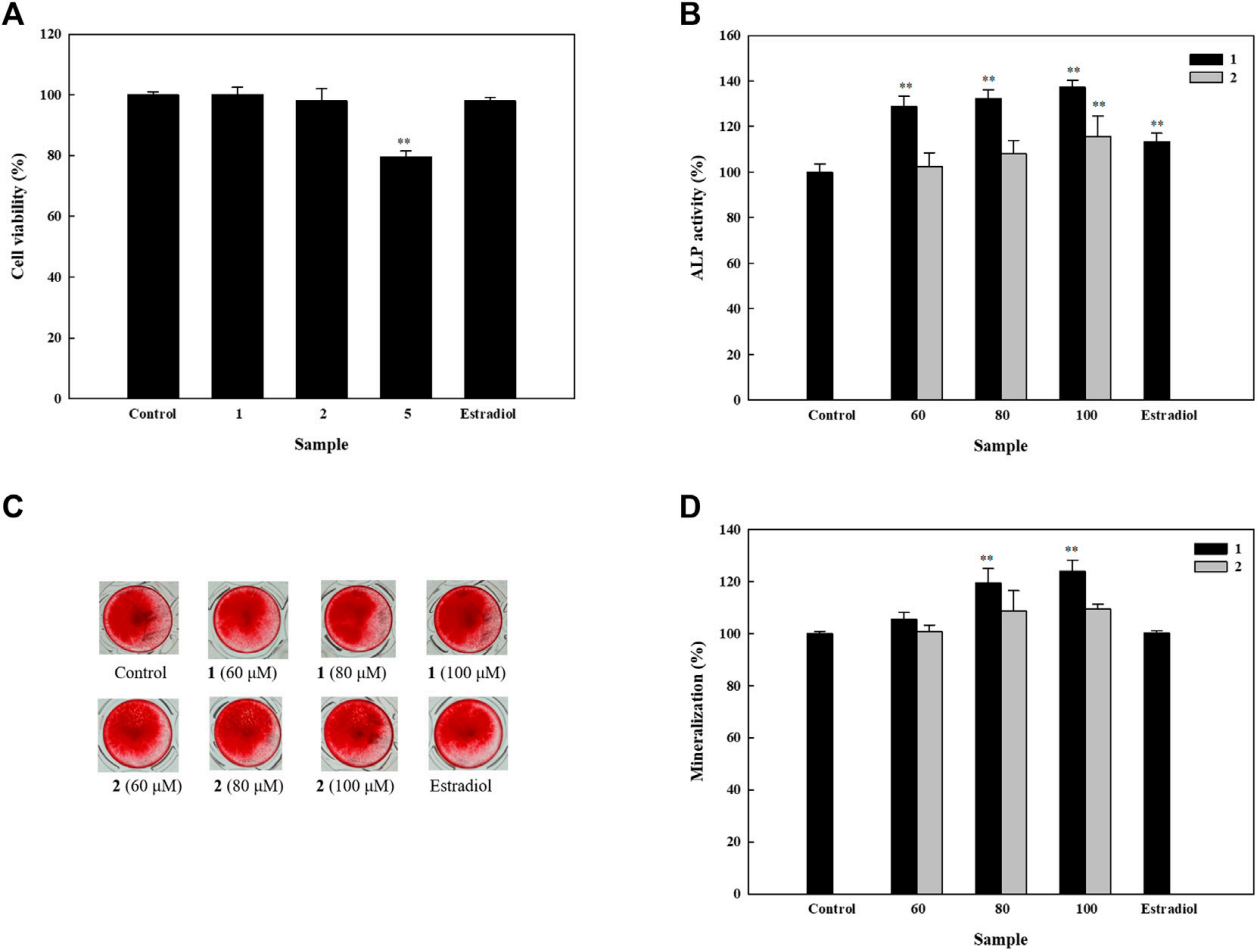
Effects of isolated compounds on cell viability, alkaline phosphatase (ALP) activity, and mineralization in human osteoblast cells. Cells were seeded on 96-well plates for **(A)** cell viability and **(B)** ALP activity assays. Samples were added after 24 h of seeding, which was followed by performing detection assays after 5 days. To assess mineralization, cells were seeded in 48-well plates, and after 4 days, drugs were added alternatively using osteoblast differentiation medium and enhancers. An alizarin red S assay was performed after 11 days. **(C)** Detection of mineral deposition after using alizarin red S stain. **(D)** Quantification of mineral deposition. Experiments were carried out in triplicate. * *p* < 0.05 and ** *p* < 0.01; data are presented as the mean ± standard deviation. **1**, astragalin; **2**, kaempferol 3-*O*-*β*-d-apiofuranosyl-(1→6)-*β*-d-glucopyranoside; and **5**, 2,5-dimethoxy-3-*O*-*β*-d-glucopyranosyl cinnamic alcohol.

Upon analyzing the effects of isolated compounds **1**, **2**, and **5** on the cell viability of HOb cells, results showed that compounds **1** and **2** had no significant effect on the cell viability of HOb cells (Figure 3A). However, isolated compounds **3** and **4** were extensively studied in our previous research, in which they were isolated from a different plant (Mao et al., 2014). Thus, we only studied the effects of compounds **1** and **2** on bone formation in HOb cells.

### Effects of Isolated Compounds on Alkaline Phosphatase Activity and Mineralization of HOb Cells

In order to identify the osteogenic potential of compounds **1** and **2**, we first studied their effects on ALP activity. Compound **1** increased ALP activity in a dose-dependent manner, with 60, 80, and 100 μM, increasing ALP activity to 128.8, 132.3, and 137.3% respectively. In contrast, compound **2** significantly increased ALP activity up to 115.8% at 100 µM (Figure 3B). These results indicate that both compounds can induce ALP activity and therefore, their activities on mineral deposition were examined in HOb cells. We found that compound **1** significantly increased mineral deposition to 119.5 and 124.0% at 80 and 100 μM, respectively. Similarly, compound **2** at 80 and 100 μM, increased mineral deposition up to 108.8 and 109.6% respectively (Figures 3C,D). Given the significance of ALP as an inducer of mineralization, the difference in the level of mineralization between **1** and **2**, could be, due in part, to their difference in ALP activity.

### Effects of the Isolated Active Compounds on Expressions of ESRs in HOb Cells

**FIGURE 4:**
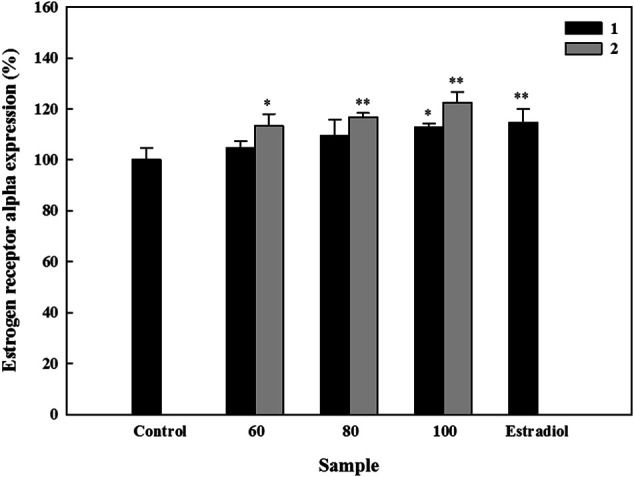
Effects of active compounds on the expression of estrogen receptor (ESR)-α in human osteoblast cells. Cells were seeded in 96-well plates, and samples were added 24 h later. After five days, expression of ESR-α was detected using an ELISA kit according to the manufacturer’s instructions. Experiments were carried out in triplicate. * *p* < 0.05 and ** *p* < 0.01; data are presented as the mean ± standard deviation. **1**, astragalin and **2**, kaempferol 3-*O*-*β*-d-apiofuranosyl-(1→6)-*β*-d-glucopyranoside.

ERs play essential roles in the process of bone formation (Khosla et al., 2012). On analyzing the effects of compounds **1** and **2** on ESR-α and -β, we observed that neither compound **1** nor **2** had a significant effect on ESR-β (Supplementary Figure S6). However, both compounds markedly increased the ESR-α expression in the HOb cells. Thus, we asked whether the compounds could exhibit a dose-dependent effect on ESR-α expression. While Compound **1** exhibited a dose-dependent increase, with 60, 80, and 100 µM increasing ESR-α expression up to 113.5, 116.8, and 122.5% respectively, compound **2** only increased ESR-α expression to 112.8% at 100 µM (Figure 4). Similar to the ALP and mineralization results, these results showed that compound **1** is more effective in inducing ESR-α that compound **2**.

### Effects of the Isolated Active Compounds on the Molecular Biomarkers in HOb Cells

**FIGURE 5:**
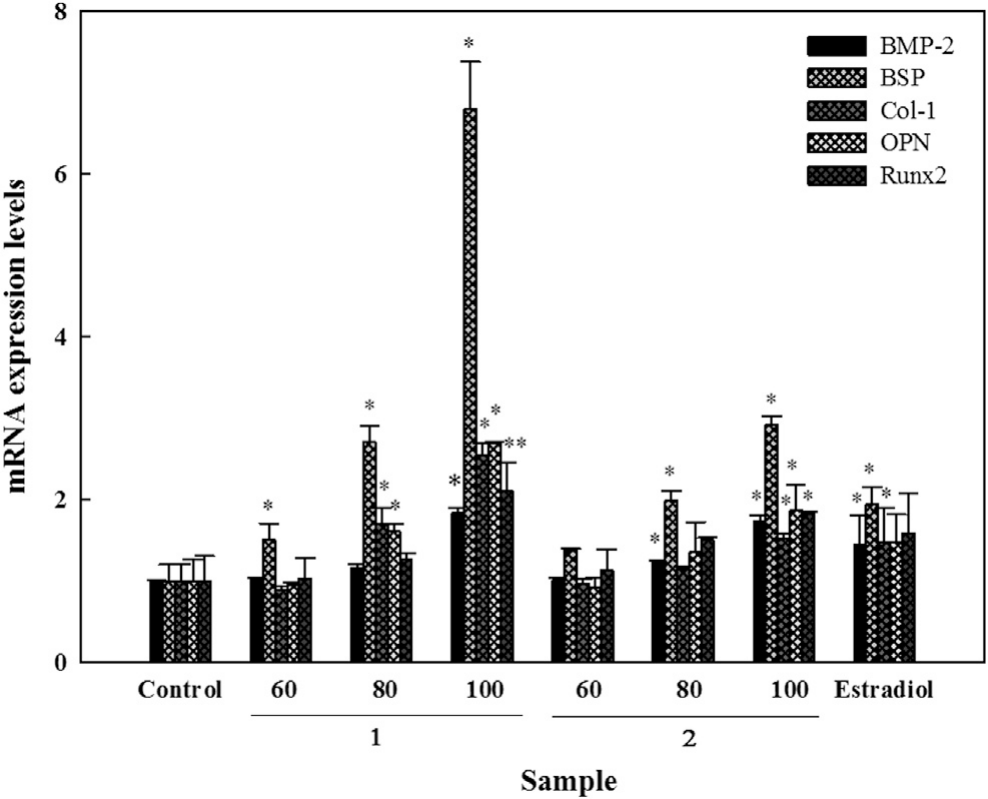
Effects of active compounds on mRNA expression levels of bone formation-related genes in human osteoblast cells. Cells were seeded in 6 cm dishes, and after 24 h, samples were added using fresh osteoblast differentiation medium. Following this, cells were collected for RNA isolation and cDNA generation. mRNA expression levels were detected using a UPL-probe system, and data are depicted as multiples. Experiments were carried out in triplicate. * *p* < 0.05 and ** *p* < 0.01; data are presented as the mean ± standard deviation. **1**, astragalin and **2**, kaempferol 3-*O*-β-d-apiofuranosyl-(1→6)-β-d-glucopyranoside.

Genetic markers which play essential roles in the process of osteoblast differentiation and bone formation were examined under the influence of compounds **1** and **2**. For BMP-2, compound **2** induced increased its messenger (m)RNA expression in a dose-dependent manner, which were up to 1.3- and 1.7-fold at 80 and 100 μM, respectively. In contrast, compound **1** only induced a significant increase of up to 1.8-fold at 100 µM. Increases in mRNA expression of BSP were induced in a dose-dependent manner by both compounds; compound **1** increased expression levels up to 1.5-, 2.7-, and 6.8-fold, while increases by compound **2** were up to 1.3-, 2.0-, and 3.0-fold at 60, 80, and 100 μM, respectively. Compound **1** also increased the mRNA expressions of Col-1 (by 1.7- and 2.5-fold) and OPN (by 1.6- and 2.7-fold) at 80 and 100 μM, respectively. Besides, it also increased the expression level of Runx2 up to 2.1-fold at 100 µM. Significant increases of up to 1.5-, 1.9-, and 1.8-fold were seen in expression levels of Col-1, OPN, and Runx2 at 100 µM of compound **2**, respectively (Figure 5).

### Effects of the Isolated Compounds on RANKL-Induced Osteoclastogenesis

**FIGURE 6:**
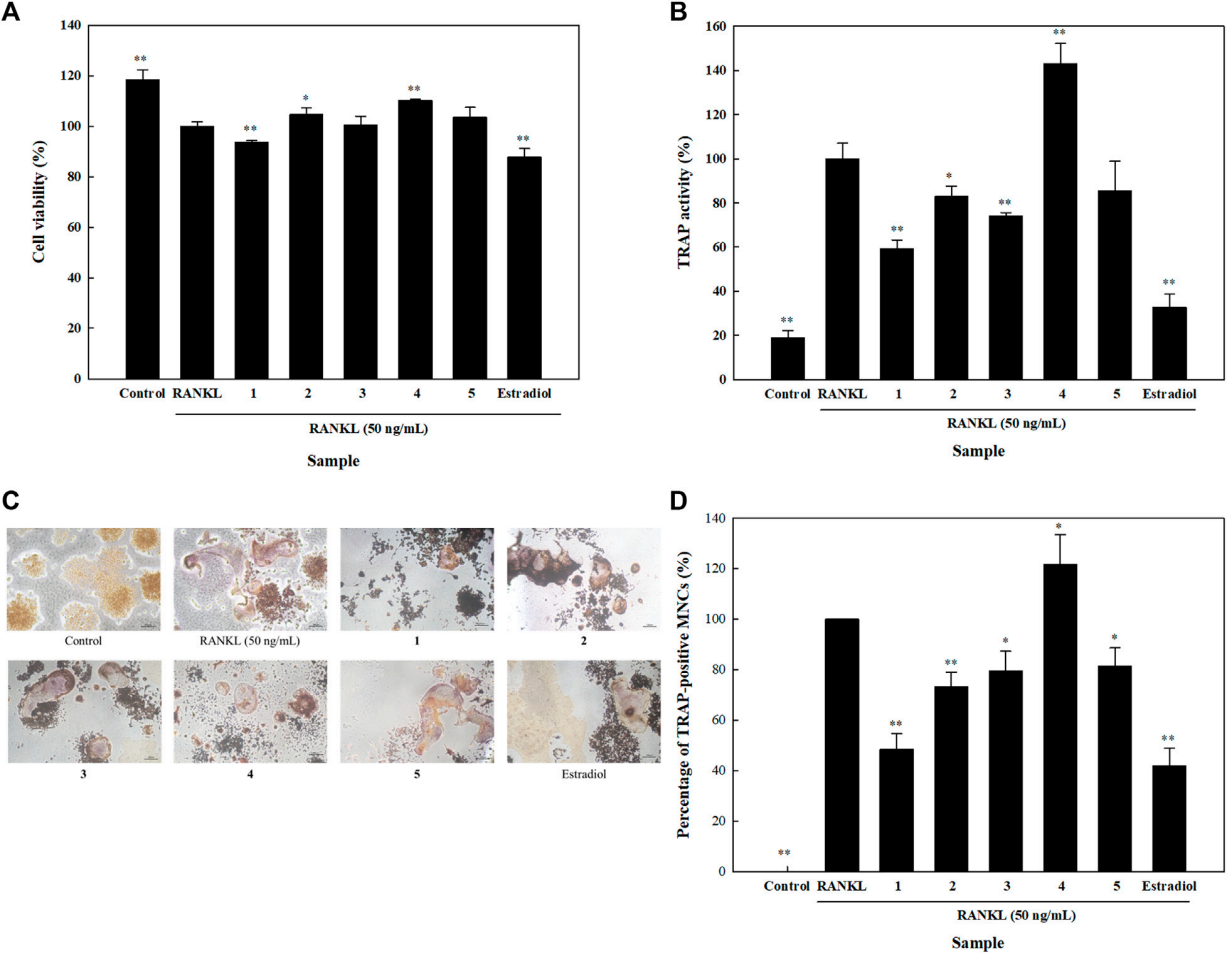
Effects of isolated compounds on cell viability, tartrate-resistant acid phosphatase (TRAP) activity and TRAP staining in receptor activator of nuclear factor-κB ligand (RANKL)-induced osteoclasts. Cells were seeded on 96-well plates for (**A**) cell viability and **(B)** TRAP activity assays. RANKL and samples were added 24 h after seeding, and detection assays were performed 5 days later. **(C)** For TRAP staining, cells were seeded in 24-well plates, and RANKL and samples were alternatively added. A leukocyte acid phosphatase kit was utilized after 5 days. **(D)** Quantification of TRAP-positive multinucleated cells (MNCs). Experiments were carried out in triplicate. * p < 0.05 and ** *p* < 0.01; data are presented as the mean ± standard deviation. **1**, astragalin; **2**, kaempferol 3-*O*-*β*;-d-apiofuranosyl-(1→6)-*β*-d-glucopyranoside; **3**, adenosine; **4**, tryptophan; and **5**, 2,5-dimethoxy-3-*O*-*β*-d-glucopyranosyl cinnamic alcohol.

Bone resorption is an important step in bone remodeling. In order to fully elucidate the roles of the isolated compounds on the process of bone remodeling, their effects on osteoclastogenesis were studied by evaluating their influence on the cell viability and TRAP activity of RANKL-induced osteoclasts. Results showed that none of the five isolated compounds had a significant effect on the viability of RANKL-induced osteoclasts. Compared to the RANKL group, cell viability was higher than 90% in all the other study groups (Figure 6A). Compounds **1**, **2**, **3**, and **5** significantly inhibited TRAP activity by 40.8, 17.1, 25.9, and 14.5%, respectively (Figure 6B). Moreover, TRAP staining and the numbers of TRAP-positive MNCs of isolated compounds were assessed. Compared to the RANKL group, compounds **1**, **2**, **3**, and **5** reduced the number of TRAP-positive MNCs by 51.6, 26.8, 20.5, and 18.6%, respectively (Figures 6C,D). These results indicated that isolated compounds **1**, **2**, **3**, and **5** attenuated RANKL-mediated osteoclast differentiation and formation.

## Discussion

Natural products are an essential source of secondary metabolites, and various activities of these compounds have been explored. Several drugs were designed from natural compounds to fight diseases, such as the discovery of aspirin from *Salix alba* L, artemisinin from *Artemisia annua*, and others (Weathers et al., 2014; Aleebrahim-Dehkordy et al., 2017). There are numerous plants that possess various medicinal properties, and after initial screening, we found that WT displays properties of promoting bone formation (Supplementary Table S1). Thus, we chose certain biomarkers to explore which part of the plant is highly active, and then we further carried out a series of chromatographic purification and bio-guided isolation to obtain pure compounds which were studied. In the present study, we utilized 70% acetone to perform extraction of *W. taiwanensis*, then fractionation using Diaion gel. Among the isolated compounds, adenosine (**3**) and tryptophan (**4**) were previously studied by our group (Mao et al., 2014). So we extensively explored the effects of astragalin (**1**) and kaempferol 3-*O*-*β*-d-apiofuranosyl-(1→6)-*β*-d-glucopyranoside (**2**) on osteoblastic and osteoclastic cells to elucidate their effects on the process of bone formation and understand the underlying mechanisms.

As mentioned before, the underlying idea of using two antagonist cell lines to confirm a singular outcome is to identify a strategy that can later be developed and used for designing better therapeutic agents. ALP is regarded as one of the most common biomarkers of osteoblast differentiation, and both compounds **1** and **2** significantly induce ALP activity (Figure 3B). ALP plays an essential role in mineral deposition, and upon analyzing the effects of compounds **1** and **2** on mineralization, we found that both compounds increased mineralization (Figure 3C). However, compound **1** exhibited a stronger effect on mineralization than compound **2**. Genes like BSP and OPN, which are characterized as small integrin-binding ligand *N*-linked glycoproteins, play key roles in bone development by facilitating mineral nucleation, mineralization, and cell adhesion; they also possess a collagen-binding domain (Zurick et al., 2013). Our results showed that both compounds **1** and **2** significantly increased BSP and OPN levels, with compound **1** yielding a more significant increase in the expression of these genes than compound **2**, which is consistent with our mineralization results (Figure 5). *Runx2* belongs to the runt domain gene family, and it is essential for the development and formation of bone. It plays a vital role in the maturation of osteoblasts by regulating expressions of matrix proteins such as osteocalcin, OPN and Col-1 (Haxaire et al., 2016). Our results showed that both compounds **1** and **2** could increase the mRNA expression of Runx2, OPN, and Col-1 in HOb cells, indicating the bone formation potentials of these compounds.

Several phytochemicals have the capacity to mimic activities of human proteins, and flavonoids are well-known to have estrogenic activity (Baker and Lathe, 2018); so we studied their effects on ERs in HOb cells. Compounds **1** and **2** were both able to significantly increase ESR-α expression; however, we found that compound **2** was more active than compound **1**, suggesting that compound **2** might be a stronger phytoestrogen. TRAP is expressed by osteoclasts, and it initiates the dephosphorylation of matrix phosphoproteins like OPN and BSP; thus, it is considered to be a specific histochemical marker for osteoclasts (Blumer et al., 2012). We found that compounds **1**, **2**, **3**, and **5** significantly inhibited TRAP activity. As mentioned earlier, TRAP targets OPN and BSP, and compounds **1** and **2** were also able to increase their mRNA levels, which means that they both can enhance osteoblast activity while decreasing osteoclast activity at non-cytotoxic levels.

Astragalin (**1**), also known as kaempferol-3-*O*-glucoside, was found to possess antioxidant (Cho et al., 2014) and anti-inflammatory activities (Korb-Pap et al., 2016). It was also reported to be effective against acute ischemia-reperfusion injury in Sprague-Dawley rats *via* attenuation of intracellular oxidative stress and apoptosis, and was found to suppress expressions of malondialdehyde (MDA), tumor necrosis factor-α, and interleukin-6 (Qu et al., 2016). A study carried out on *Caenorhabditis elegans* demonstrated that astragalin **1**) was able to decrease 6-hydroxydopamine-induced neurodegeneration. It was found to reduce transcription levels of the proapoptotic gene, *elg-1*, which is associated with neuronal death alongside decreases in ROS and MDA levels and inhibition of lipid peroxidation (Li et al., 2016). Compound **1** was reported to be effective against hyperglycemia and diabetic retinopathy by alleviating the effects of high glucose in the blood (Ke et al., 2012). Several studies showed the anticancer effect of compound **1**, as it was observed to significantly inhibit the proliferation of cancer cell lines such as HL-60 leukemia cells, HaCaT skin cancer cells, and A549 lung cancer cells (Burmistrova et al., 2011; You et al., 2017). In a separate study, astragalin **1**) isolated from *Cuscuta chinensis*, displayed estrogenic activity in osteoblast-like UMR-106 cells, and enhanced their cell proliferation (Yang et al., 2011). In that study, astragalin (**1**) was unable to induce a significant increase in ALP activity; however, astragalin (**1**) may promote osteoblastic differentiation in MC3T3-E1 cells *via* BMP and MAPK pathways and also promote bone formation in ovariectomized-induced osteoporotic mouse (Liu et al., 2019). In our study, compound **1** significantly increased ALP activity, by up to 128.8%. The possible reasons for this discrepancy could be due to the different cell lines used, as in our study, we used human osteoblasts. Many studies have shown several bioactivities of compound **1**, however, our study is the first to show that it has anti-osteoporotic potential not only *via* increasing bone formation markers but also through suppressing osteoclastogenesis. Due to the limited amount of the isolated compound **1**, in the future, this compound could be a potential candidate for chemical synthesis and molecular docking of its novel derivatives (Chen et al., 2019). These molecules can be then used to evaluate the bone remodeling potential using *in vivo* models.

We investigated the structure-activity relationship of two isolated flavonol glycosides. Astragalin (kaempferol 3-*O*-*β*-d-glucopyranoside, **1**) and kaempferol 3-*O*-*β*-d-apiofuranosyl-(1→6)-*β*-d-glucopyranoside (**2**) are both glycosides of kaempferol, resulting from various modifications. Kaempferol was found to be very effective at enhancing bone formation, and studies showed that after treating ovariectomized rats with kaempferol, the microarchitectural parameters and bone mineral density significantly increased (Nowak et al., 2017; Zhao et al., 2019). Kaempferol was found to increase mRNA levels of BSP, OSX, and Runx2 in UMR106 cells (Yang et al., 2010). In our study, both active compounds, **1** and **2**, were able to induce significantly increases the mRNA expression levels of BSP and Runx2 in a dose-dependent manner. However, compound **1** was more efficient at enhancing the mRNA expression levels of these genes. Thus, it is possible to deduce that the higher osteogenesis of compound **1** was due to the absence of an apiofuranosyl group. As glucocorticoids are significant causal agents of bone loss, kaempferol was reported to attenuate glucocorticoid-induced bone loss in adult female rats (Adhikary et al., 2018), and also promoted bone formation (Zhao et al., 2019). Besides, kaempferol and its derivatives were reported to induce bone formation, and also reported to have remarkable effects on bone resorption (Suvarna et al., 2018). In our study on analyzing the effects of the isolated compounds on osteoclasts, we found that compounds **1**, **2**, and **3** significantly decreased TRAP activity at non-cytotoxic concentration, indicating that these compounds can equally decrease osteoclast activity. Likewise, compound **1** exhibited higher osteoclastogenesis was due to the absence of an apiofuranosyl group.

## Conclusion

In this study, the active compounds isolated from 70% acetone extract of *W. taiwanensis*, astragalin (**1**) and kaempferol 3-*O*-*β*-d-apiofuranosyl-(1→6)-*β*-d-glucopyranoside (**2**), enhanced bone formation while simultaneously impeding bone resorption, as evidenced by their effects on the markers of bone formation and bone resorption. Our findings, therefore, pinpoint *W. taiwanesis* as a natural bioactive agent with potent anti-osteoprosis attributes, deserving further evaluation for development as an anti-osteoporosis treatment.

## Data Availability

The original contributions presented in the study are included in the article/Supplementary Material, further inquiries can be directed to the corresponding author.

## Abbreviations

ALP, alkaline phosphatase; BMP-2, bone morphogenetic protein-2; BSP, bone sialoprotein; Col-1, type I collagen; ESR, estrogen receptor; HObs, primary human osteoblasts; HPLC, high-performance liquid chromatography; OPG, osteoprotegerin; OPN, osteopontin; OSX, osterix; RANKL, receptor of nuclear factor-κB; TRAP, tartrate-resistant acid phosphatase; WT, *Wikstroemia taiwanensis* Chang.

## References

[B1] Adhikary S. Choudhary D. Ahmad N. Karvande A. Kumar A. Banala V. T. (2018). Dietary Flavonoid Kaempferol Inhibits Glucocorticoid-Induced Bone Loss by Promoting Osteoblast Survival. Nutrition 53, 64–76. 10.1016/j.nut.2017.12.003 29655780

[B2] Aleebrahim-Dehkordy E. Nasri H. Baradaran A. Nasri P. Tamadon M. R. Hedaiaty M. (2017). Medicinal Plants, Effective Plant Compounds (Compositions) and Their Effects on Stomach Cancer. Int. J. Prev. Med. 8, 96. 10.4103/ijpvm.IJPVM_4_17 29184647 PMC5686923

[B3] Baker M. E. Lathe R. (2018). The Promiscuous Estrogen Receptor: Evolution of Physiological Estrogens and Response to Phytochemicals and Endocrine Disruptors. J. Steroid Biochem. Mol. Biol. 184, 29–37. 10.1016/j.jsbmb.2018.07.001 30009950

[B4] Blumer M. J. F. Hausott B. Schwarzer C. Hayman A. R. Stempel J. Fritsch H. (2012). Role of Tartrate-Resistant Acid Phosphatase (TRAP) in Long Bone Development. Mech. Develop. 129, 162–176. 10.1016/j.mod.2012.04.003 PMC3419267 22579636

[B5] Burmistrova O. Quintana J. Díaz J. G. Estévez F. (2011). Astragalin Heptaacetate-Induced Cell Death in Human Leukemia Cells Is Dependent on Caspases and Activates the MAPK Pathway. Cancer Lett. 309, 71–77. 10.1016/j.canlet.2011.05.018 21658841

[B6] Chen L.-Y. Chen I.-S. Peng C.-F. (2012). Structural Elucidation and Bioactivity of Biflavonoids from the Stems of *Wikstroemia Taiwanensis* . Int. J. Mol. Sci. 13, 1029–1038. 10.3390/ijms13011029 22312302 PMC3269736

[B7] Chen L. Z. Yao L. Jiao M. M. Shi J. B. Tan Y. Ruan B. F. (2019). Novel Resveratrol-Based Flavonol Derivatives: Synthesis and Anti-inflammatory Activity *In Vitro* and *In Vivo* . Eur. J. Med. Chem. 175, 114–128. 10.1016/j.ejmech.2019.05.004 31077997

[B8] Chen X. Liu X. H. Duan N. Zhu G. Schwarz E. M. Xie C. (2018). Osteoblast-osteoclast Interactions. Eur. J. Med. Chem. 59, 99–107. 10.1080/03008207.2017.1290085 PMC5612831 28324674

[B9] Cho I.-H. Gong J.-H. Kang M.-K. Lee E.-J. Park J. H. Y. Park S.-J. (2014). Astragalin Inhibits Airway Eotaxin-1 Induction and Epithelial Apoptosis through Modulating Oxidative Stress-Responsive MAPK Signaling. BMC Pulm. Med. 14, 122. 10.1186/1471-2466-14-122 25069610 PMC4118077

[B10] Crockett J. C. Rogers M. J. Coxon F. P. Hocking L. J. Helfrich M. H. (2011). Bone Remodelling at a Glance. J. Cel Sci. 124, 991–998. 10.1242/jcs.063032 21402872

[B11] Deng S. Deng Z. Fan Y. Peng Y. Li J. Xiong D. (2009). Isolation and Purification of Three Flavonoid Glycosides from the Leaves of *Nelumbo nucifera* (Lotus) by High-Speed Counter-current Chromatography. J. Chromatogr. B 877, 2487–2492. 10.1016/j.jchromb.2009.06.026 19592314

[B12] Eriksen E. F. (2010). Cellular Mechanisms of Bone Remodeling. Rev. Endocr. Metab. Disord. 11, 219–227. 10.1007/s11154-010-9153-1 21188536 PMC3028072

[B13] Foo L. Y. Lu Y. Molan A. L. Woodfield D. R. Mcnabb W. C. (2000). The Phenols and Prodelphinidins of white clover Flowers. Phytochemistry 54, 539–548. 10.1016/s0031-9422(00)00124-2 10939359

[B14] Haxaire C. Haÿ E. Geoffroy V. (2016). Runx2 Controls Bone Resorption through the Down-Regulation of the Wnt Pathway in Osteoblasts. Am. J. Pathol. 186, 1598–1609. 10.1016/j.ajpath.2016.01.016 27083516

[B15] Hsu F.-L. Chou C.-J. Chang Y.-C. Chang T.-T. Lu M.-K. (2006). Promotion of Hyphal Growth and Underlying Chemical Changes in *Antrodia Camphorata* by Host Factors from *Cinnamomum Camphora* . Int. J. Food Microbiol. 106, 32–38. 10.1016/j.ijfoodmicro.2005.07.003 16219379

[B16] Imtiyaz Z. Wang Y.-F. Lin Y.-T. Liu H.-K. Lee M.-H. (2019). Isolated Compounds from *Turpinia Formosana* Nakai Induce Ossification. Int. J. Mol. Sci. 20, 3119. 10.3390/ijms20133119 PMC6651545 31247918

[B17] Ke M. Hu X.-Q. Ouyang J. Dai B. Xu Y. (2012). The Effect of Astragalin on the VEGF Production of Cultured Müller Cells under High Glucose Conditions. Biomed. Mater. Eng. 22, 113–119. 10.3233/BME-2012-0696 22766709

[B18] Khong A. Forestieri R. Williams D. E. Patrick B. O. Olmstead A. Svinti V. (2012). A Daphnane Diterpenoid Isolated from *Wikstroemia Polyantha* Induces an Inflammatory Response and Modulates miRNA Activity. PloS one 7, e39621. 10.1371/journal.pone.0039621 22761847 PMC3383676

[B19] Khosla S. Oursler M. J. Monroe D. G. (2012). Estrogen and the Skeleton. Trends Endocrinol. Metab. 23, 576–581. 10.1016/j.tem.2012.03.008 22595550 PMC3424385

[B20] Kim T.-Y. Park N.-J. Jegal J. Choi S. Lee S. W. Hang J. (2019). Chamaejasmine Isolated from *Wikstroemia Dolichantha* Diels Suppresses 2,4-Dinitrofluoro-Benzene-Induced Atopic Dermatitis in SKH-1 Hairless Mice. Biomolecules 9, 697. 10.3390/biom9110697 PMC6921031 31694198

[B21] Korb-Pap A. Bertrand J. Sherwood J. Pap T. (2016). Stable Activation of Fibroblasts in Rheumatic Arthritis-Causes and Consequences. Rheumatology 55, ii64–ii67. 10.1093/rheumatology/kew347 27856663

[B22] Lee M. Phillips R. S. (1992). Fluorine Substituent Effects for Tryptophan in13C Nuclear Magnetic Resonance. Magn. Reson. Chem. 30, 1035–1040. 10.1002/mrc.1260301102

[B23] Li H. Shi R. Ding F. Wang H. Han W. Ma F. (2016). Astragalus Polysaccharide Suppresses 6-Hydroxydopamine-Induced Neurotoxicity in *Caenorhabditis elegans* . Oxidative Med. Cell Longevity 2016, 4856761. 10.1155/2016/4856761 PMC5112302 27885333

[B24] Li S.-F. Jiao Y.-Y. Zhang Z.-Q. Chao J.-B. Jia J. Shi X.-L. (2018). Diterpenes from Buds of *Wikstroemia Chamaedaphne* Showing Anti-hepatitis B Virus Activities. Phytochemistry 151, 17–25. 10.1016/j.phytochem.2018.01.021 29631103

[B25] Li Y.-M. Zhu L. Jiang J.-G. Yang L. Wang D.-Y. (2009). Bioactive Components and Pharmacological Action of *Wikstroemia Indica* (L.) C. A. Mey and its Clinical Application. Curr. Pharm. Biotechnol. 10, 743–752. 10.2174/138920109789978748 19939213

[B26] Liu L. Wang D. Qin Y. Xu M. Zhou L. Xu W. (2019). Astragalin Promotes Osteoblastic Differentiation in MC3T3-E1 Cells and Bone Formation *In Vivo* . Front. Endocrinol. 10 (10), 228. 10.3389/fendo.2019.00228 PMC6476984 31040823

[B27] Mao Y. W. Lin R. D. Hung H. C. Lee M. H. (2014). Stimulation of Osteogenic Activity in Human Osteoblast Cells by Edible *Uraria Crinita* . J. Agric. Food Chem. 62, 5581–5588. 10.1021/jf5012177 24785825

[B28] Moyroud E. Strazewski P. (1999). l-Ribonucleosides from L-Xylose. Tetrahedron 55, 1277–1284. 10.1016/S0040-4020(98)01119-3

[B29] Niedzwiedzki T. Filipowska J. (2015). Bone Remodeling in the Context of Cellular and Systemic Regulation: the Role of Osteocytes and the Nervous System. J. Mol. Endocrinol. 55, R23–R36. 10.1530/JME-15-0067 26307562

[B30] Nowak B. Matuszewska A. Nikodem A. Filipiak J. Landwójtowicz M. Sadanowicz E. (2017). Oral Administration of Kaempferol Inhibits Bone Loss in Rat Model of Ovariectomy-Induced Osteopenia. Pharmacol. Rep. 69, 1113–1119. 10.1016/j.pharep.2017.05.002 29031689

[B31] Owen R. Reilly G. C. (2018). *In Vitro* models of Bone Remodelling and Associated Disorders. Front. Bioeng. Biotechnol. 6, 134. 10.3389/fbioe.2018.00134 30364287 PMC6193121

[B32] Qian S.-J. Zhang Y.-H. Li G.-D. (2020). The Complete Chloroplast Genome of a Medicinal Plant, *Wikstroemia Chamaedaphne* (Thymelaeaceae). Mitochondrial DNA B 5, 648–649. 10.1080/23802359.2019.1711228 PMC7748548 33366686

[B33] Qu D. Han J. Ren H. Yang W. Zhang X. Zheng Q. (2016). Cardioprotective Effects of Astragalin against Myocardial Ischemia/reperfusion Injury in Isolated Rat Heart. Oxidative Med. Cell Longevity 2016, 8194690. 10.1155/2016/8194690 PMC4695676 26788251

[B34] Suvarna V. Sarkar M. Chaubey P. Khan T. Sherje A. Patel K. (2018). Bone Health and Natural Products- an Insight. Front. Pharmacol. 9, 981. 10.3389/fphar.2018.00981 30283334 PMC6157411

[B35] Weathers P. J. Towler M. Hassanali A. Lutgen P. Engeu P. O. (2014). Dried-leafArtemisia Annua: A Practical Malaria Therapeutic for Developing Countries?. World J. Pharmacol. 3, 39–55. 10.5497/wjp.v3.i4.39 25678989 PMC4323188

[B36] Wu B. Takahashi T. Kashiwagi T. Tebayashi S.-i. Kim C.-S. (2007). New Flavonoid Glycosides from the Leaves of *Solidago Altissima* . Chem. Pharm. Bull. 55, 815–816. 10.1248/cpb.55.815 17473477

[B37] Wu Z.-B. Liu Y. Tian S.-S. Wen C. (2014). Chemical Constituents of the Stem Bark of *Fraxinus Rhynchophylla* . Chem. Nat. Compd. 49, 1162–1163. 10.1007/s10600-014-0850-y

[B38] Yan L. Lu L. Hu F. Shetti D. Wei K. (2019). Piceatannol Attenuates RANKL-Induced Osteoclast Differentiation and Bone Resorption by Suppressing MAPK, NF-κB and AKT Signalling Pathways and Promotes Caspase3-Mediated Apoptosis of Mature Osteoclasts. R. Soc. Open Sci. 6, 190360. 10.1098/rsos.190360 31312498 PMC6599799

[B39] Yang L. Chen Q. Wang F. Zhang G. (2011). Antiosteoporotic Compounds from Seeds of *Cuscuta Chinensis* . J. Ethnopharmacology 135, 553–560. 10.1016/j.jep.2011.03.056 21463675

[B40] Yang L. Takai H. Utsunomiya T. Li X. Li Z. Wang Z. (2010). Kaempferol Stimulates Bone Sialoprotein Gene Transcription and New Bone Formation. J. Cel. Biochem. 110, 1342–1355. 10.1002/jcb.22649 20564228

[B41] Yao H. Yuan Z. Wei G. Chen C. Duan J. Li Y. (2017). Thevetiaflavone from *Wikstroemia Indica* Ameliorates PC12 Cells Injury Induced by OGD/R *via* Improving ROS-Mediated Mitochondrial Dysfunction. Mol. Med. Rep. 16, 9197–9202. 10.3892/mmr.2017.7712 28990067

[B42] Yao H. Zhang W. Wu H. Yang M. Wei P. Ma H. (2019). Sikokianin A from *Wikstroemia Indica* Protects PC12 Cells against OGD/R-Induced Injury *via* Inhibiting Oxidative Stress and Activating Nrf2. Nat. Product. Res. 33, 3450–3453. 10.1080/14786419.2018.1480019 29806503

[B43] You O. H. Shin E. A. Lee H. Kim J.-H. Sim D. Y. Kim J. H. (2017). Apoptotic Effect of Astragalin in Melanoma Skin Cancers via Activation of Caspases and Inhibition of Sry-Related HMg-Box Gene 10. Phytother. Res. 31, 1614–1620. 10.1002/ptr.5895 28809055

[B44] Zhao J. Wu J. Xu B. Yuan Z. Leng Y. Min J. (2019). Kaempferol Promotes Bone Formation in Part *via* the mTOR Signaling Pathway. Mol. Med. Rep. 20, 5197–5207. 10.3892/mmr.2019.10747 31638215 PMC6854588

[B45] Zurick K. M. Qin C. Bernards M. T. (2013). Mineralization Induction Effects of Osteopontin, Bone Sialoprotein, and Dentin Phosphoprotein on a Biomimetic Collagen Substrate. J. Biomed. Mater. Res. 101A, 1571–1581. 10.1002/jbm.a.34462 PMC3619013 23161527

