# Supplementary material for: Compounds Isolated from Wikstroemia taiwanensis Regulate Bone Remodeling by Modulating Osteoblast and Osteoclast Activities
Source: Front Pharmacol. 2021 Jul 13;12:670254. doi: 10.3389/fphar.2021.670254 (PMC8327267; doi:10.3389/fphar.2021.670254)
Supplement: Supplementary file 1 [file DataSheet1.docx]

*Supplementary*

**Compounds Isolated from *Wikstroemia taiwanensis*** **Regulate Bone Remodeling by Modulating Osteoblast and Osteoclast Activities**

**Zuha Imtiyaz^1, †^, Yi-Tzu Lin^1, †^, Fang-Yu Liang^2^, Wen-Fei Chiou^3^,** **Mei-Hsien Lee^1,2,4, *^**

^1^PhD in Clinical Drug Development of Herbal Medicine, College of Pharmacy, Taipei Medical University, Taipei, Taiwan

^2^Graduate Institute of Pharmacognosy, College of Pharmacy, Taipei Medical University, Taipei, Taiwan

^3^National Research Institute of Chinese Medicine, Ministry of Health and Welfare, Taipei, Taiwan

^4^Center for Reproductive Medicine & Sciences, Taipei Medical University Hospital, Taipei, Taiwan

*** Correspondence:**

Mei-Hsien Lee

lmh@tmu.edu.tw

^†^ These authors have contributed equally to this work and share first authorship.

**Table S1 Screening the active plant extract using MG63 cells**

**Chart S1** Schematic representation of compound isolation from *W. taiwanensis*.

**Method S1** Spectral data of isolated compounds from *W. taiwanensis*.

**Figure S1** Spectra of astragalin (**1**). (A) ^1^H NMR (DMSO-*d*_6_, 500 MHz) spectrum. (B) ^13^C NMR (DMSO-*d*_6_, 125 MHz) spectrum. (C) COSY spectrum. (D) HSQC spectrum. (E) HMBC spectrum. (F) Acid hydrolysis.

**Figure S2** Spectra of kaempferol 3-*O*-*β*-d-apiofuranosyl-(1→6)-*β*-d-glucopyranoside (**2**). (A) ^1^H NMR (DMSO-*d_6_*, 500 MHz) spectrum. (B) ^13^C NMR (DMSO-*d*_6_, 125 MHz) spectrum.

**Figure S3** Spectra of adenosine (**3**). (A) ^1^H NMR (DMSO-*d*_6_, 500 MHz) spectrum. (B) ^13^C NMR (DMSO-*d*_6_, 125 MHz) spectrum. (C) COSY spectrum. (D) HSQC spectrum. (E) HMBC spectrum.

**Figure S4** Spectra of tryptophan (**4**). (A) ^1^H-NMR (500 MHz, DMSO-*d*_6_) spectrum. (B) ^13^C-NMR (125 MHz, DMSO-*d*_6_) spectrum. (C) COSY spectrum. (D) HSQC spectrum. (E) HMBC spectrum.

**Figure S5** Spectra of 2,5-dimethoxy-3-*O*-*β*-d-grucopyranosyl cinnamic alcohol (**5**). (A) ^1^H-NMR (500 MHz, DMSO-*d*_6_) spectrum. (B) ^13^C-NMR (125 MHz, DMSO-*d*_6_). (C) COSY spectrum. (D) HSQC spectrum. (E) HMBC spectrum.

**Figure S6** Effects of compounds **1** and **2** on the expression of estrogen receptor (ESR)-β.

**Table S1**

| Sample | | ALP activity (%) |
| --- | --- | --- |
| Control | 100.0 ± 1.7 |  |
| T39 | 96.1 ± 1.7 |  |
| T44 | 95.3 ± 1.8 |  |
| T46 | 95.0 ± 1.4 |  |
| T55 | 100.2 ± 1.7 |  |
| T69 | 97.7 ± 0.9 |  |
| T74 | 95.7 ± 4.0 |  |
| T84 | 103.4 ± 1.7 |  |
| T85 | 79.9 ± 3.3 |  |
| T86 | 88.6 ± 2.5 |  |

Human osteoblast-like cells (MG-63 cells) were cultured in 96-well plates and treated with extracts of various Taiwanese endemic plants for 3 days. After that their ALP activity was measured. T84 is 70% acetone extract of *Wikstroemia taiwanensis* leaves. Values are presented as mean ± SD, where 𝑛 = 6, ^∗^ *p* ≤ 0.05 and ^∗∗^ *p* ≤ 0.01 versus control.

**Chart S1**


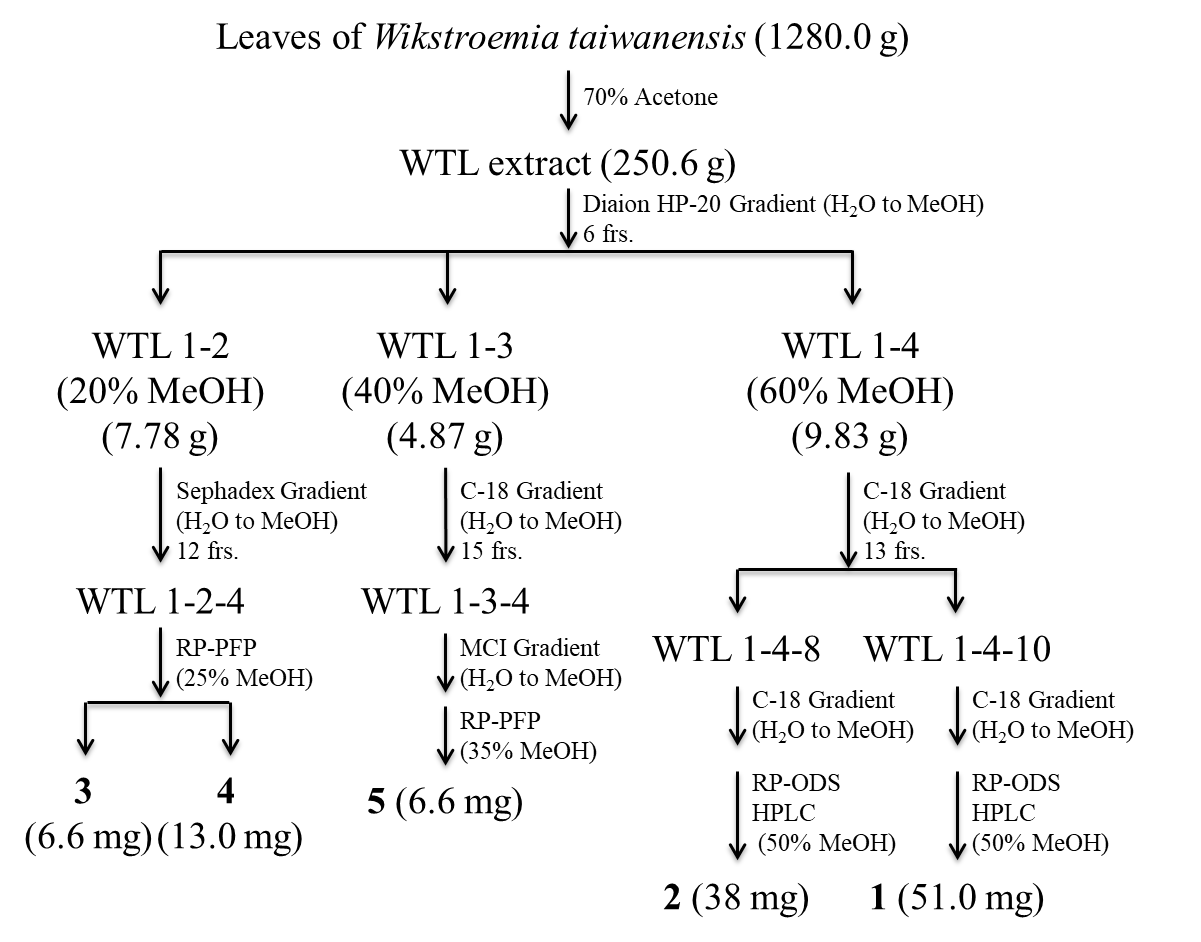


**Method S1** Spectral data of isolated compounds

Astragalin (**1**): ^1^H-NMR (500 MHz, DMSO-*d*_6_). δ_H_: 12.60 (1H, s, 5-OH), 10.88 (1H, s, 7-OH), 8.03 (2H, d, *J* = 7.0 Hz, H-2′, H-6′), 6.87 (2H, d, *J* = 7.0 Hz, H-3′, H-5′), 6.43 (1H, d, *J* = 2.1 Hz, H-8), 6.20 (1H, d, *J* = 2.1 Hz, H-6), 5.44 (1H, d, *J* = 7.5 Hz, H-1′′), 3.55 (1H, dd, *J* = 11.4, 1.4 Hz, H-6′′), 3.38 (1H, m, H-6′′), 3.19 (1H, m, H-3′′), 3.16 (1H, m, H-2′′), 3.07 (1H, m, H-5′′), 3.06 (1H, m, H-4′′). ^13^C-NMR (125 MHz, DMSO-*d*_6_). δ_C_: 177.5 (C-4), 164.2 (C-7), 161.3 (C-5), 160.0 (C-4′), 156.4 (C-9), 156.3 (C-2), 133.2 (C-3), 130.9 (C-2′, C-6′), 120.9 (C-1′), 115.1 (C-3′, C-5′), 104.0 (C-10), 100.9 (C-1′′), 98.7 (C-6), 93.7 (C-8), 77.5 (C-5′′), 76.4 (C-3′′), 74.2 (C-2′′), 69.9 (C-4′′), 60.9 (C-6′′).

Kaempferol 3-*O*-*β*-d-apiofuranosyl-(1→6)-*β*-d-glucopyranoside (**2**): ^1^H-NMR (500 MHz, DMSO-*d*_6_). δ_H_: 7.99 (2H, d, *J* = 8.9 Hz, H-2′, H-6′), 6.87 (2H, d, *J* = 8.9 Hz, H-3′, H-5′), 6.40 (1H, d, *J* = 2.1 Hz, H-8), 6.18 (1H, d, *J* = 2.1 Hz, H-6), 5.36 (1H, d, *J* = 7.6 Hz, H-1′′), 4.67 (1H, d, *J* = 2.7 Hz, H-1′′′), 3.68 (1H, d, *J* = 10 Hz, H-2′′′), 3.60 (1H, d, *J* = 9.4 Hz, H-4′′′), 3.55 (1H, m, H-6′′), 3.43 (1H, d, *J* = 9.4 Hz, H-4′′′), 3.30 (1H, m, H-6′′), 3.25 (1H, m, H-5′′), 3.22 (1H, m, H-3′′), 3.19 (1H, m, H-2′′), 3.16 (1H, s, H-5′′′), 3.05 (1H, m, H-4′′). ^13^C-NMR (125 MHz, DMSO-*d*_6_). δ_C_: 177.4 (C-4), 164.3 (C-7), 161.3 (C-5), 159.9 (C-4′), 156.6 (C-9), 156.5 (C-2), 133.1 (C-3), 130.9 (C-2′, C-6′), 120.9 (C-1′), 115.1 (C-3′, C-5′), 109.3 (C-1′′′), 104.0 (C-10), 100.9 (C-1′′), 98.8 (C-6), 93.7 (C-8), 78.7 (C-3′′′), 76.3 (C-2′′′), 75.9 (C-5′′), 75.9 (C-3′′), 74.2 (C-2′′), 73.2 (C-4′′′), 69.9 (C-4′′), 67.3 (C-6′′), 63.5 (C-5′′′).

Adenosine (**3**): ^1^H-NMR (500 MHz, DMSO-*d*_6_). δ_H_: 8.32 (1H, s, H-8), 8.12 (1H, s, H-2), 7.29 (2H, brs, NH_2_), 5.86 (1H, d, *J* = 6.2 Hz, H-1′), 5.45 (2H, brs, 2′-OH, 5′-OH), 5.20 (1H, brs, 3′-OH), 4.58 (1H, m, H-2′), 4.13 (1H, m, H-3′), 3.95 (1H, m, H-4′), 3.65 (1H, m, H-5′), 3.54 (1H, m, H-5′). ^13^C-NMR (125 MHz, DMSO-*d*_6_). δ_C_: 156.2 (C-6), 152.5 (C-2), 149.2 (C-4), 140.1 (C-8), 119.4 (C-5), 88.1 (C-1′), 86.0 (C-4′), 73.6 (C-2′), 70.8 (C-3′), 61.8 (C-5′).

Tryptophan (**4**): ^1^H-NMR (500 MHz, DMSO-*d*_6_). δ_H_: 10.89 (1H, s, NH), 7.59 (1H, d, *J* = 7.7 Hz, H-4), 7.33 (1H, d, *J* = 7.7 Hz, H-7), 7.19 (1H, d, *J* = 1.8 Hz, H-2), 7.05 (1H, t, *J* = 7.7 Hz, H-6), 6.96 (1H, t, *J* = 7.7 Hz, H-5), 3.48 (1H, dd, *J* = 9.2, 3.8 Hz, H-2′), 3.31 (1H, dd, *J* = 15.1, 3.8 Hz, H-1′), 2.96 (1H, dd, *J* = 15.1, 9.2 Hz, H-1′). ^13^C-NMR (125 MHz, DMSO-*d*_6_). δ_C_: 170.6 (C3′), 136.5 (C-8), 127.4 (C-9), 124.3 (C-2), 121.1 (C-6), 118.6 (C-4), 118.5 (C-5), 111.5 (C-7), 109.5 (C-3), 54.9 (C-2′), 27.2 (C-1′).

2,5-Dimethoxy-3-*O*-*β*-d-grucopyranosyl cinnamic alcohol (**5**): ^1^H-NMR (500 MHz, DMSO-*d*_6_). δ_H_: 6.72 (2H, s, H-4, H-6), 6.46 (1H, d, *J* = 15.9 Hz, H-7), 6.33 (1H, dt, *J* = 15.9, 5.1 Hz, H-8), 4.97 (1H, d, *J* = 3.7 Hz, H-1′), 4.10 (2H, d, *J* = 5.1 Hz, H-9), 3.76 (6H, s, 2-OCH_3_, 5-OCH_3_), 3.58 (1H, dd, *J* = 11.7, 4.9 Hz, H-6′), 3.41 (1H, dd, *J* = 11.7, 5.9 Hz, H-6′), 3.20 (1H, m, H-5′), 3.18 (1H, m, H-3′), 3.12 (1H, m, H-4′), 3.02 (1H, m, H-2′).^13^C-NMR (125 MHz, DMSO-*d*_6_). δ_C_: 152.7 (C-2, C-5), 133.8 (C-1), 132.6 (C-3), 130.1 (C-8), 128.4 (C-7), 104.4 (C-4, C-6), 102.5 (C-1′), 77.2 (C-2′), 76.5 (C-3′), 74.1 (C-5′), 69.9 (C-4′), 61.4 (C-9), 60.8 (C-6′), 56.3 (2-OCH_3_, 5-OCH_3_).

**Figure S1 (A)**


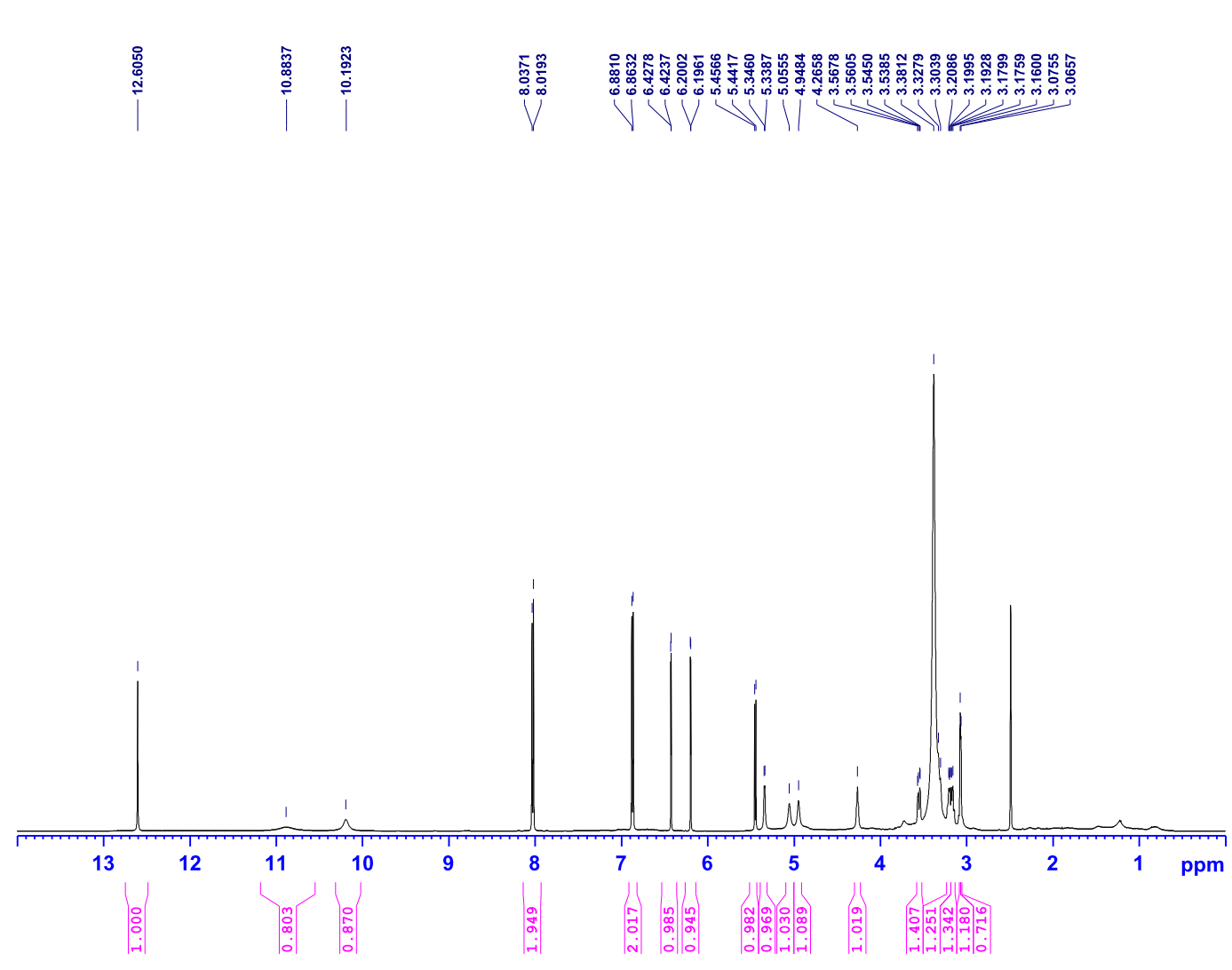


**(B)**


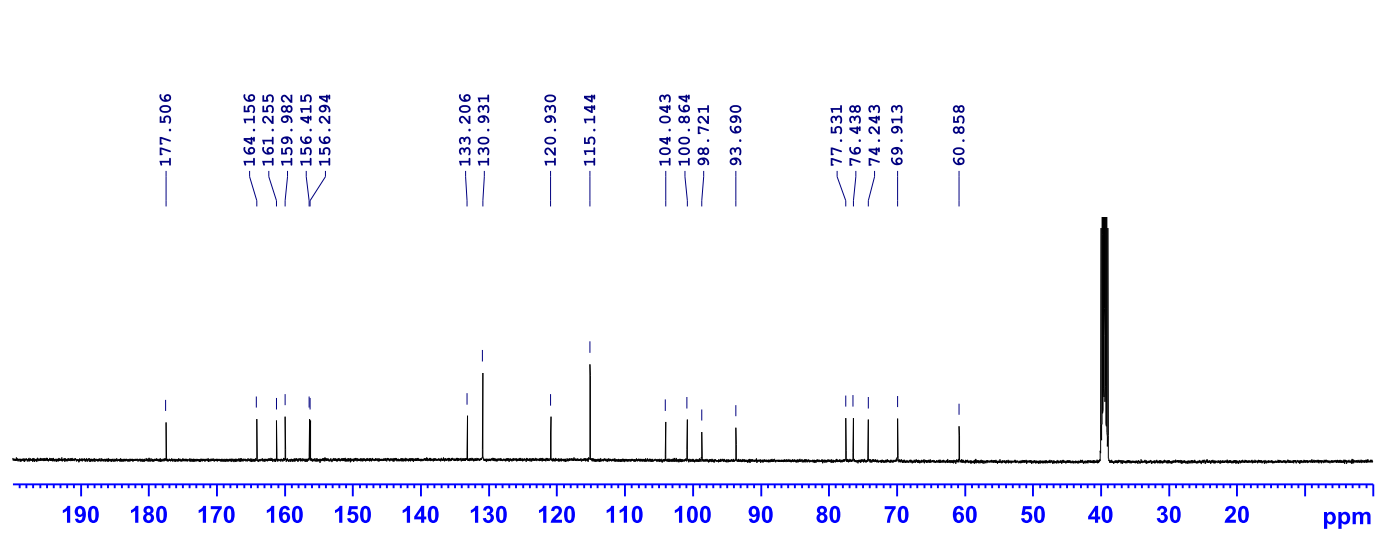


**(C)**


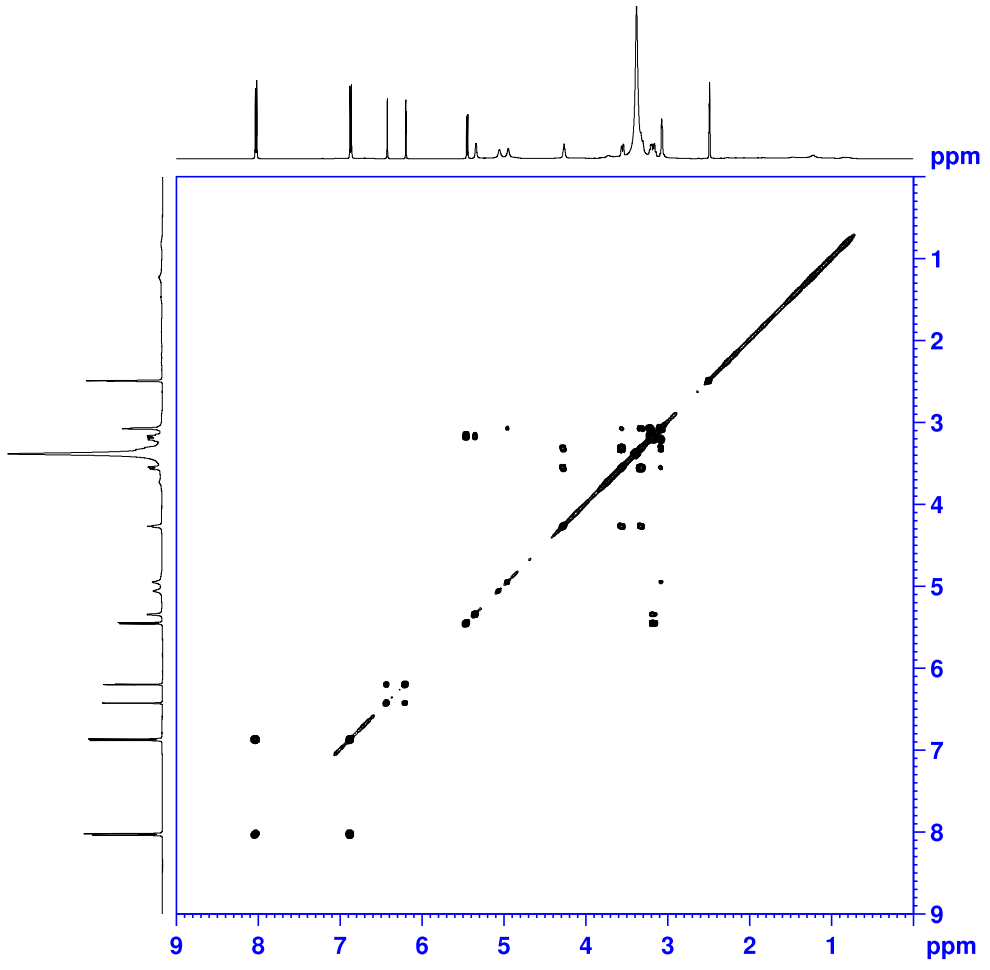


**(D)**


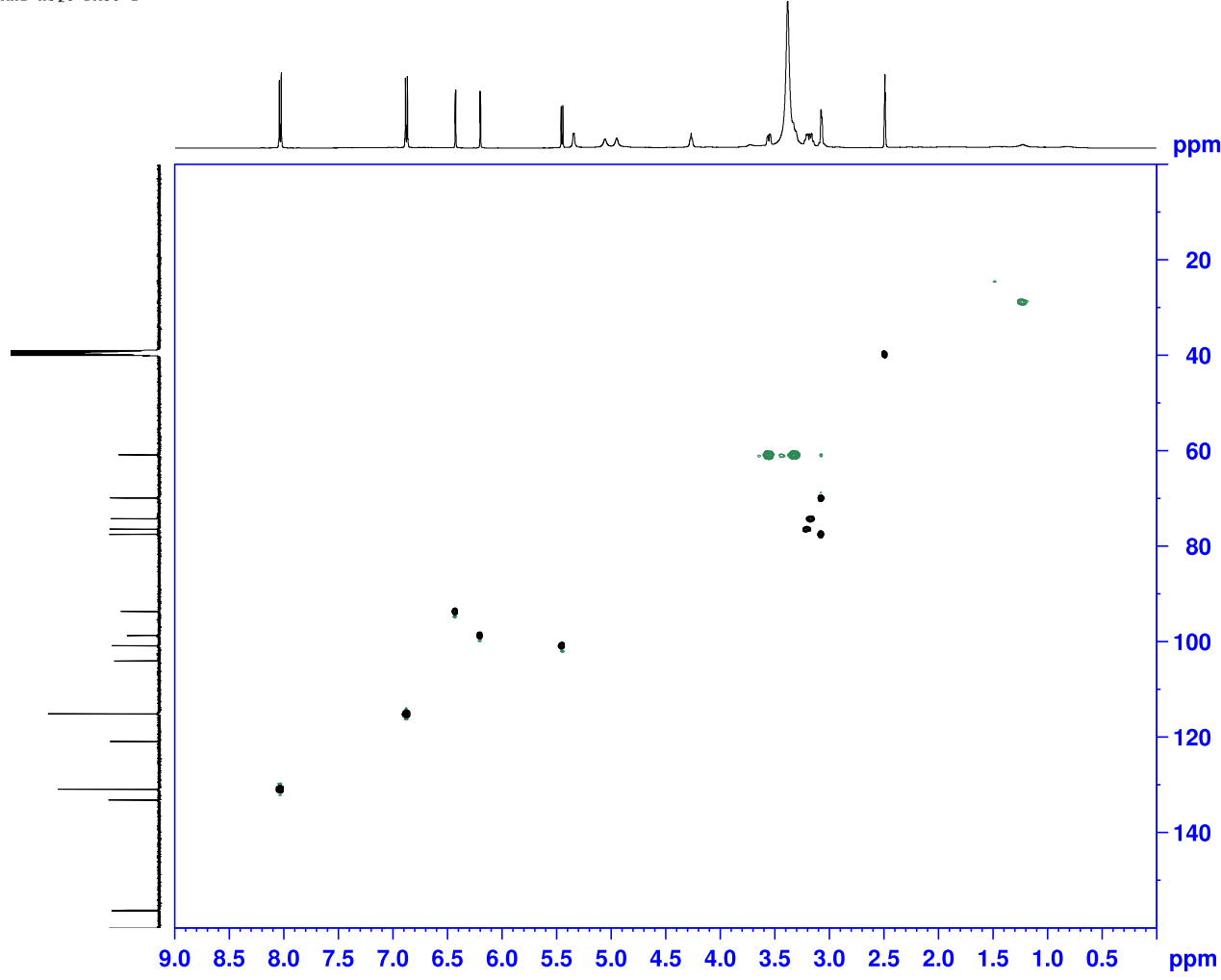


**(E)**


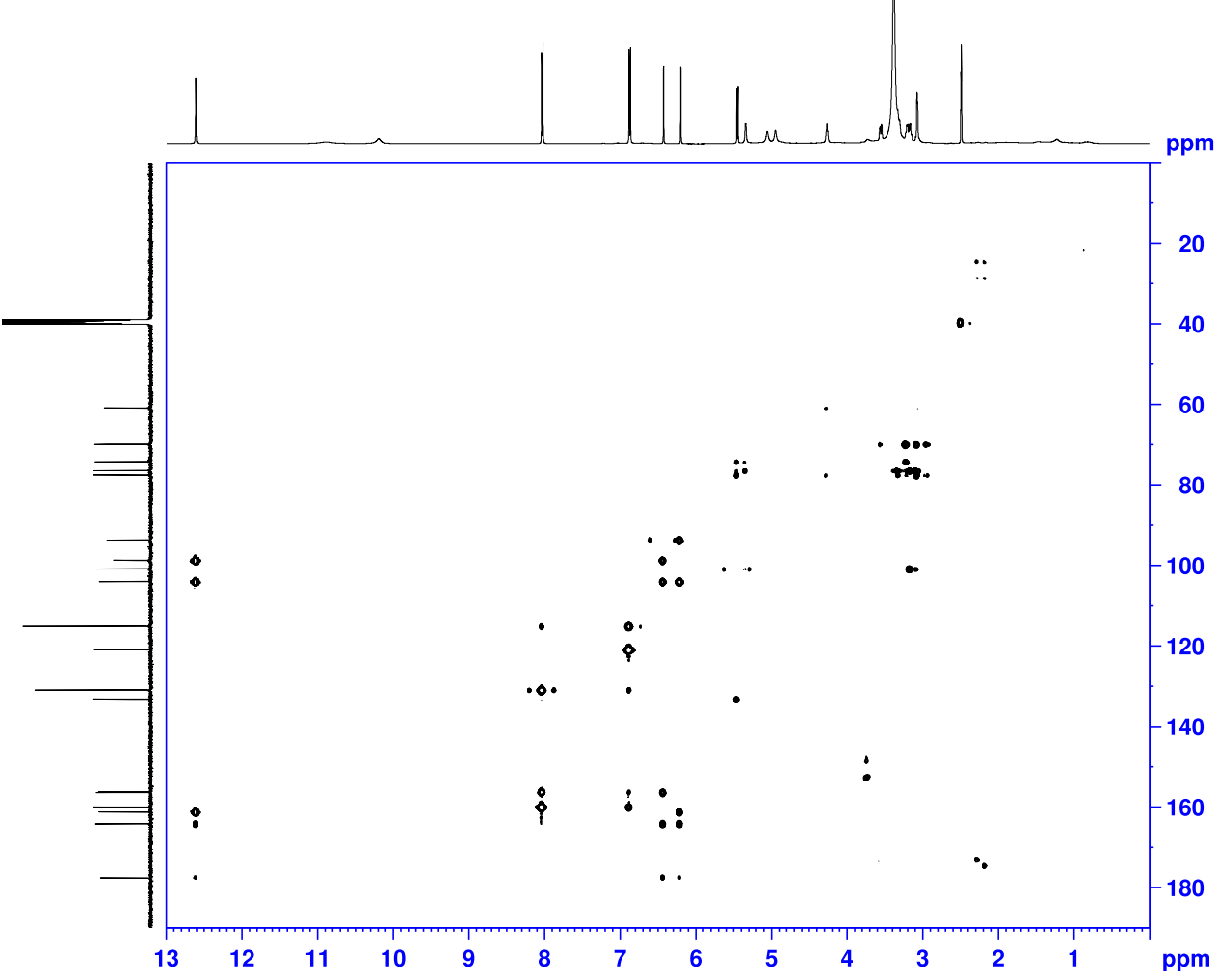


**(F)**


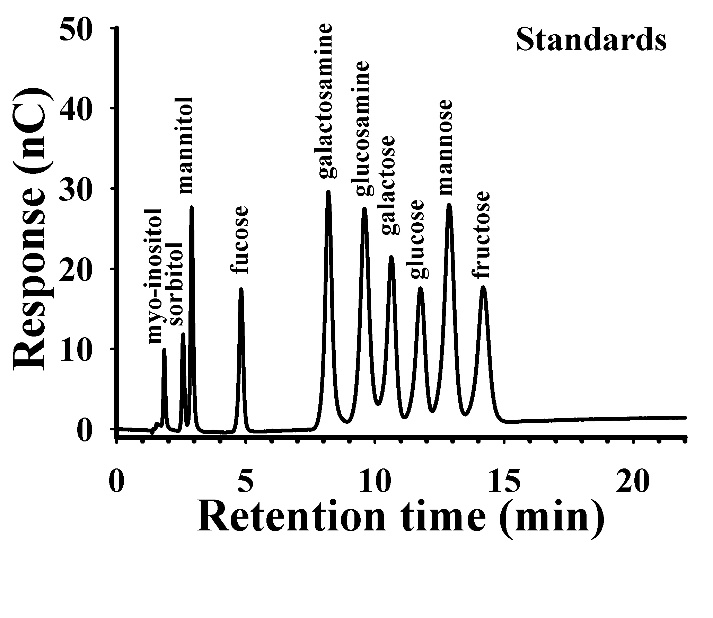


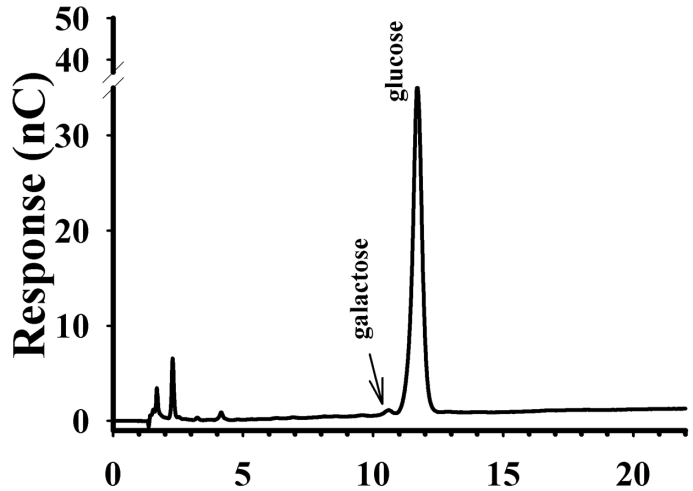


**Figure S2 (A)**


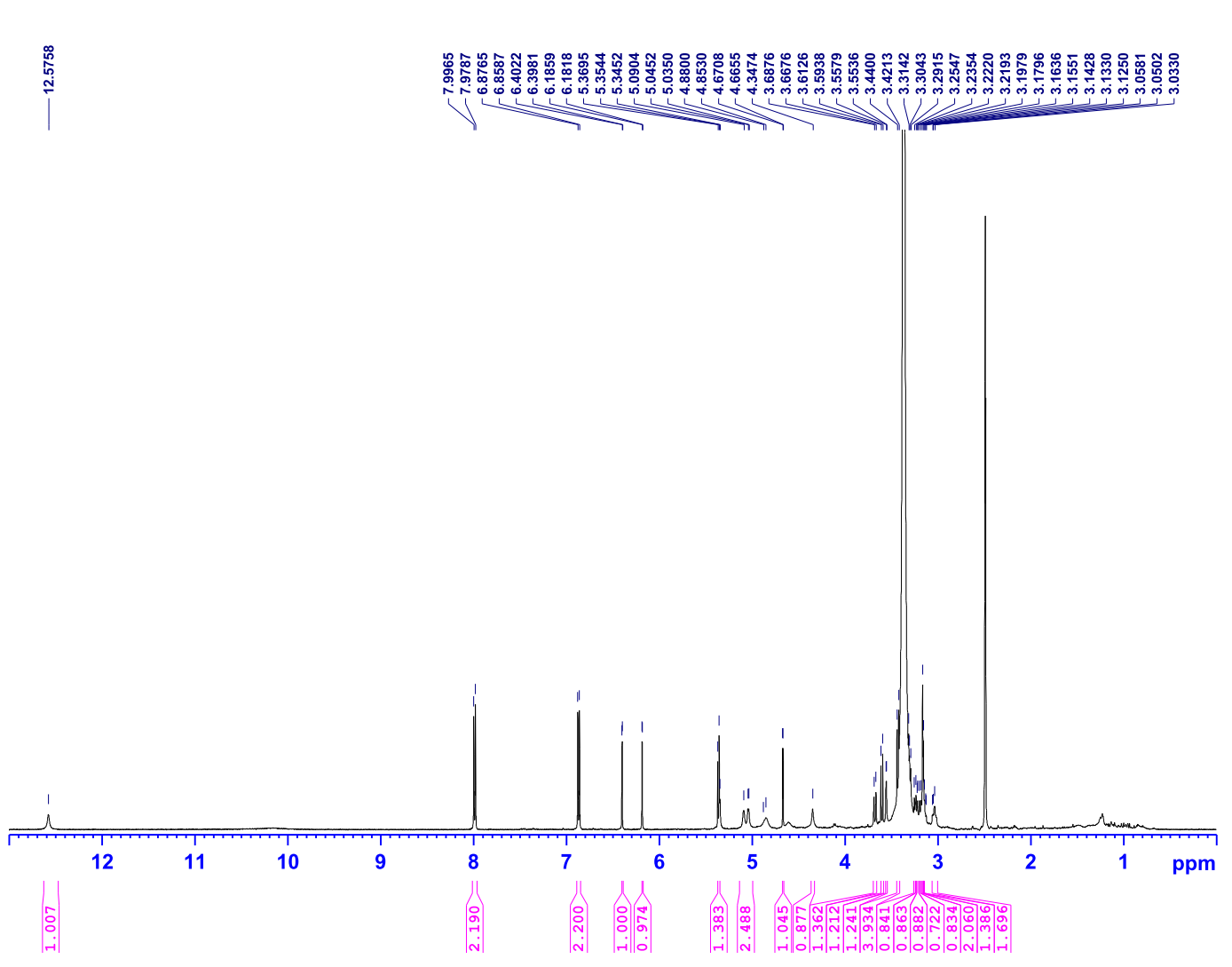


**(B)**


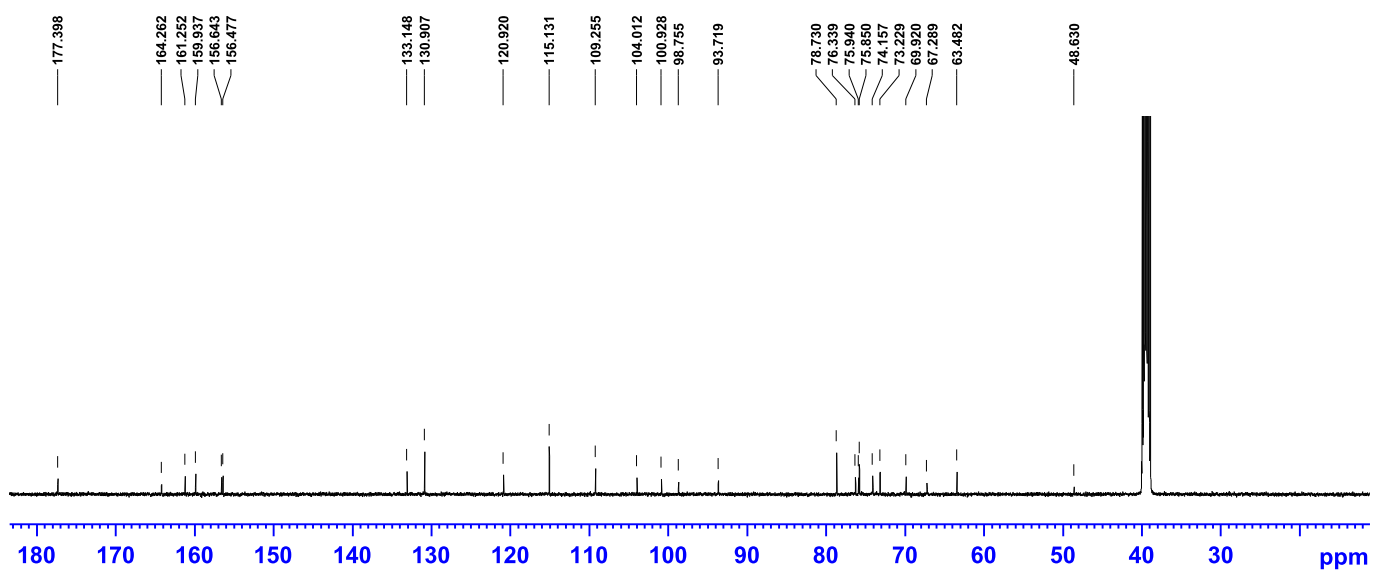


**Figure S3 (A)**


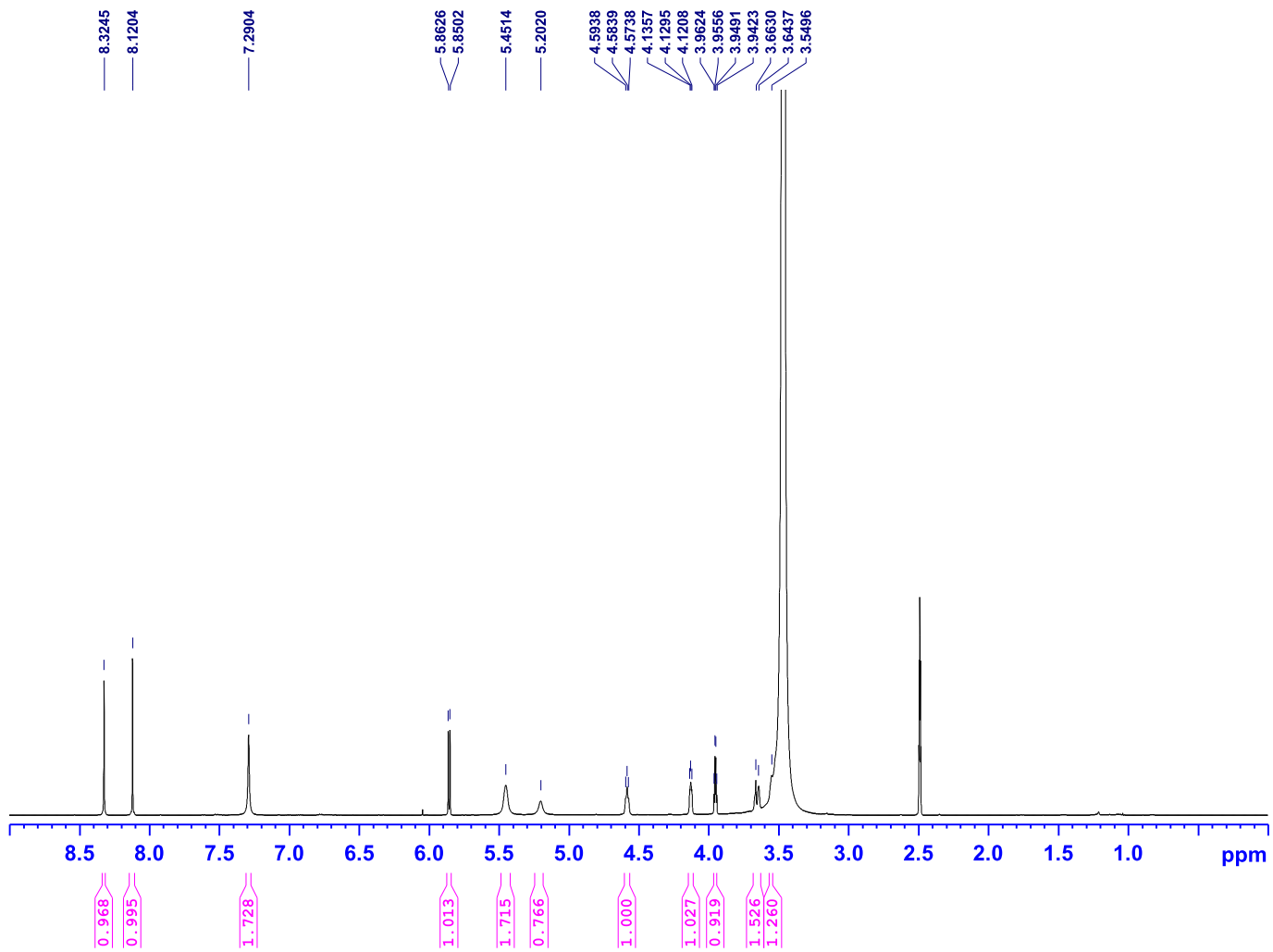


**(B)**


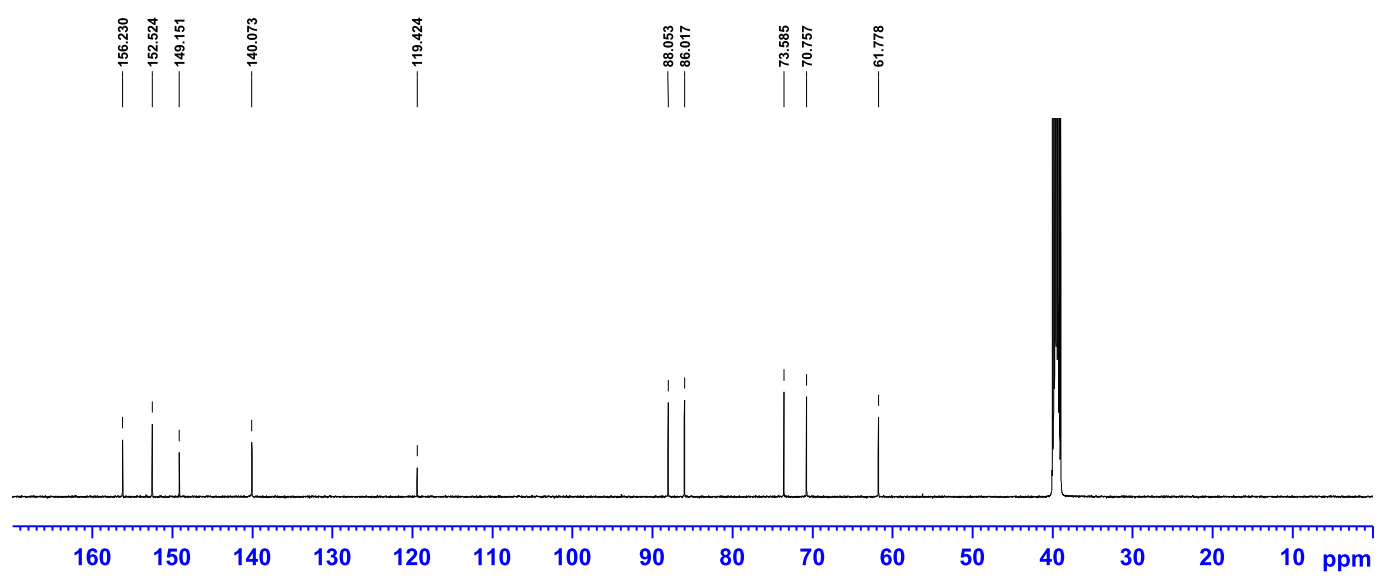


**(C)**


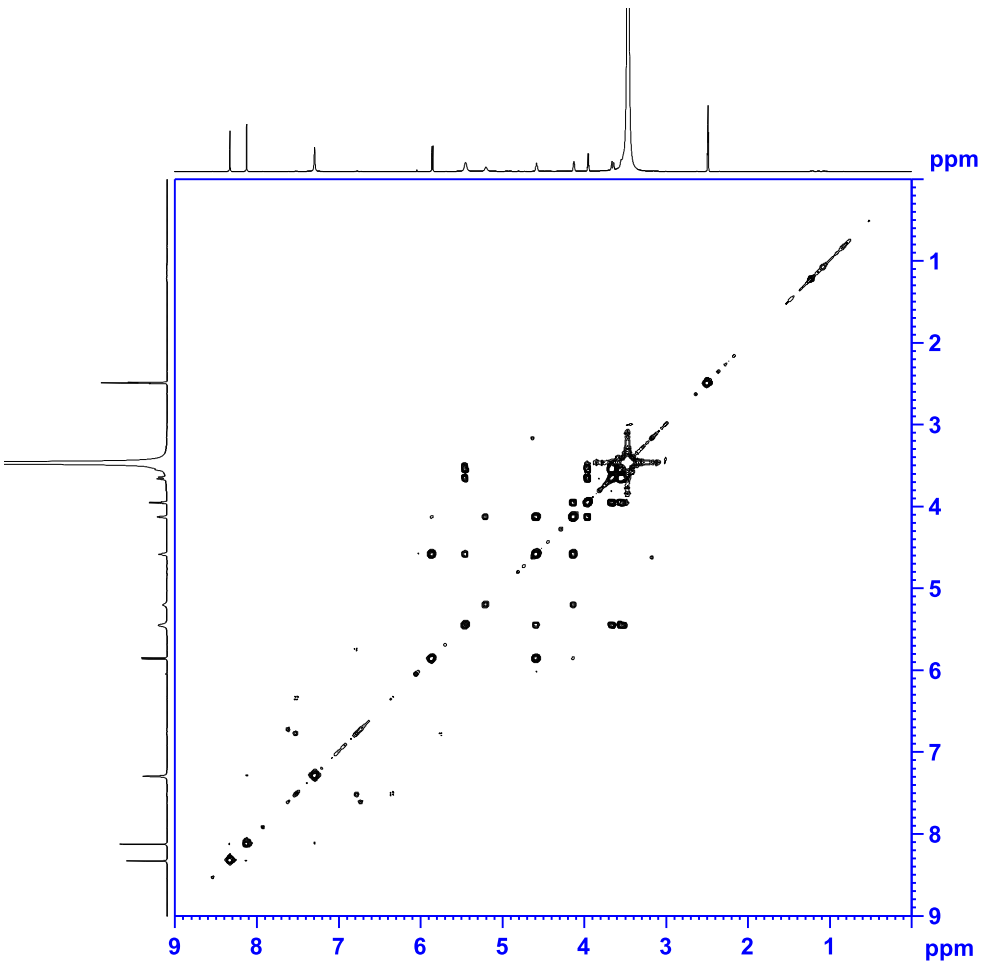


**(D)**


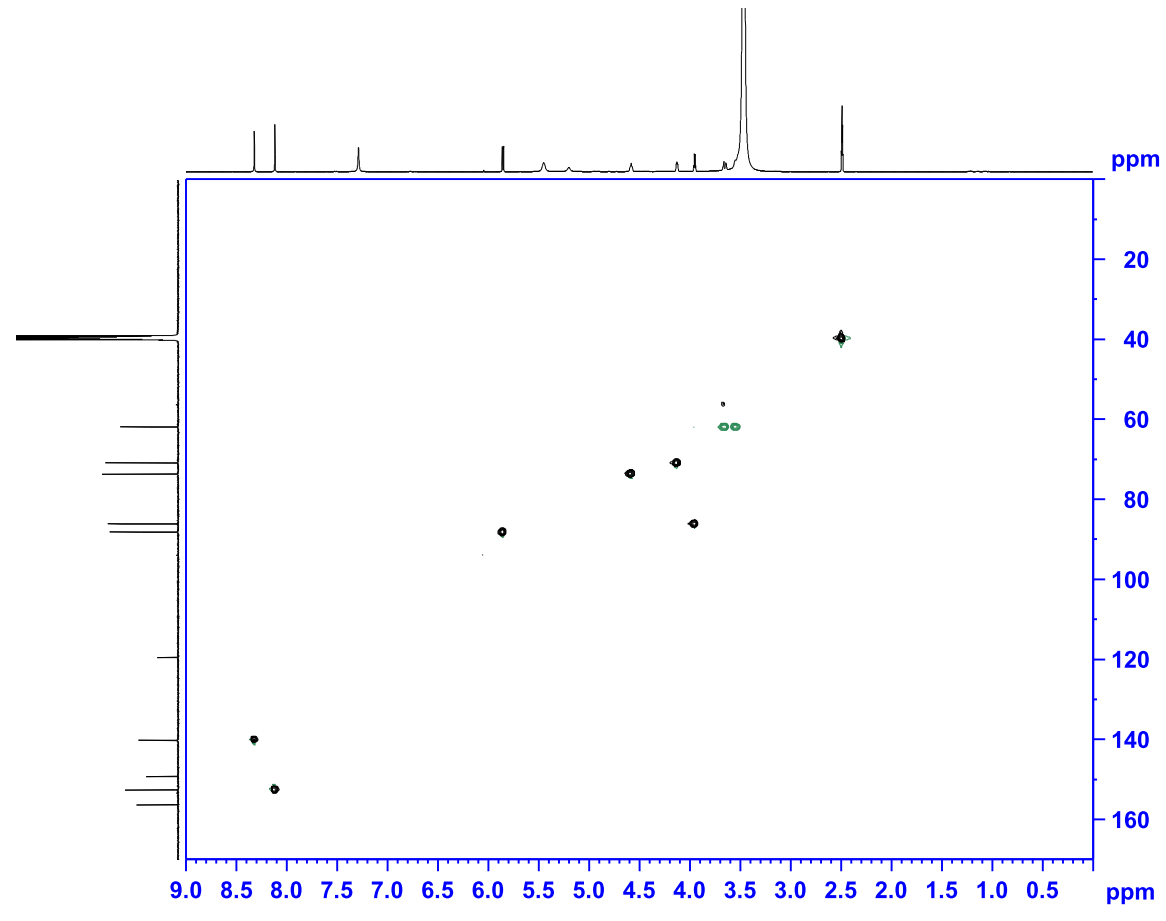


**(E)**


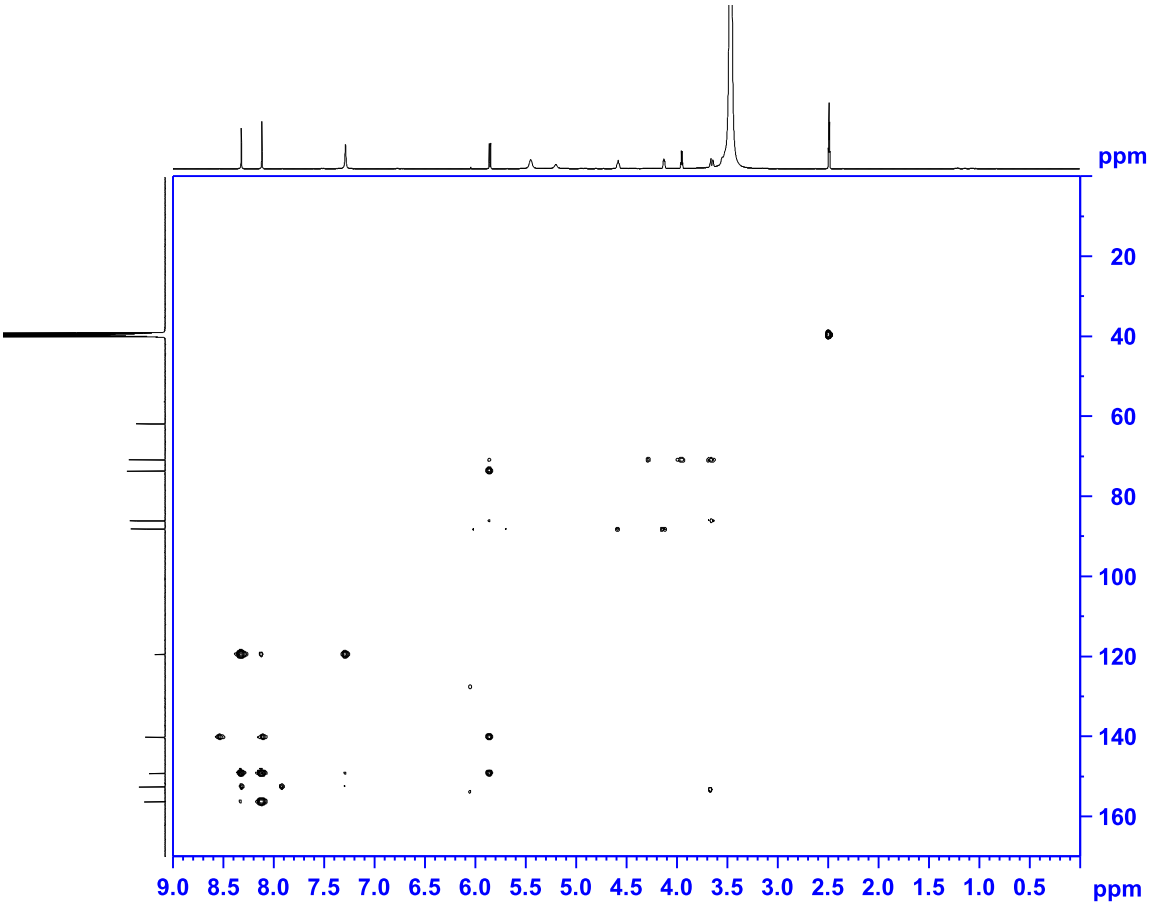


**Figure S4 (A)**


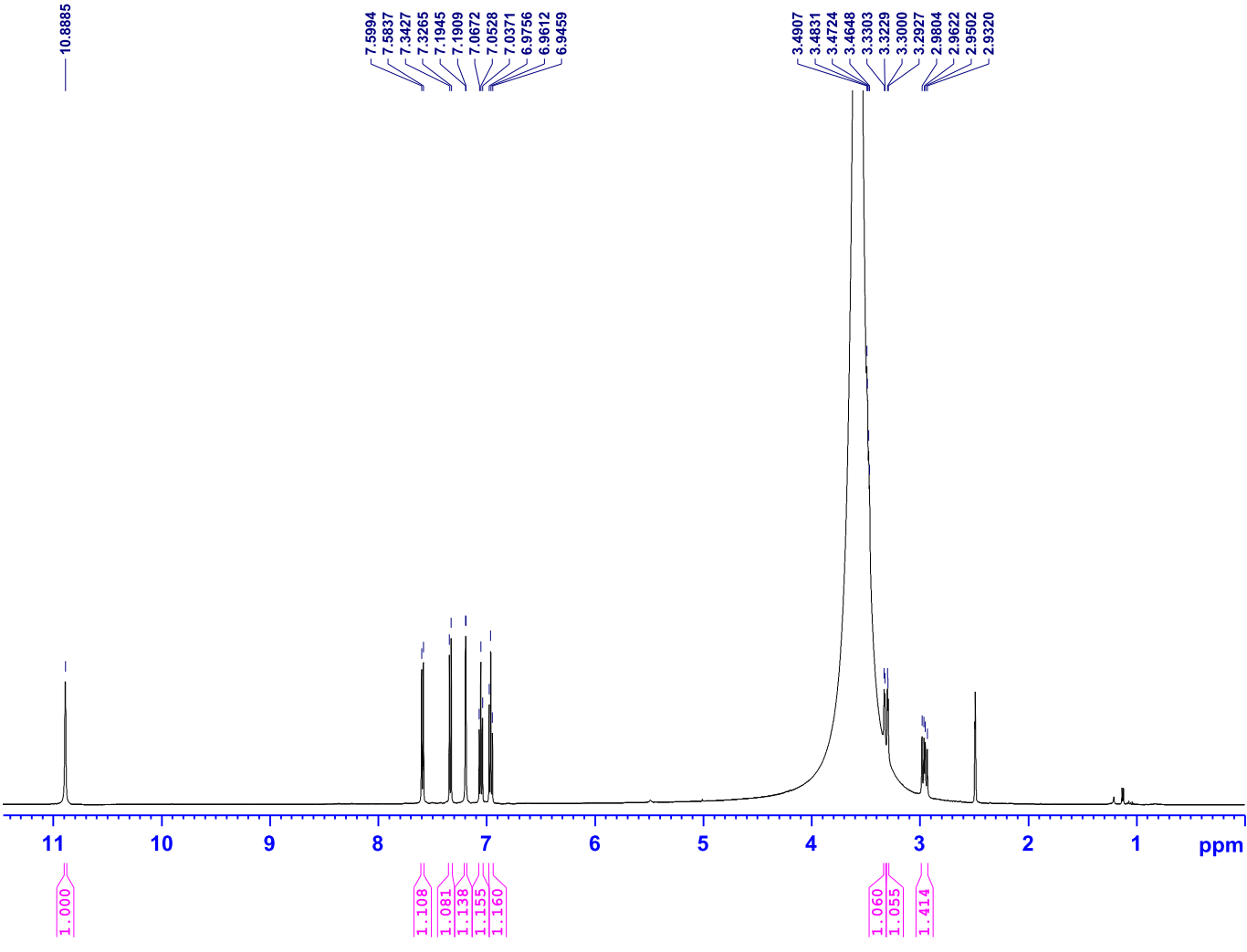


**(B)**


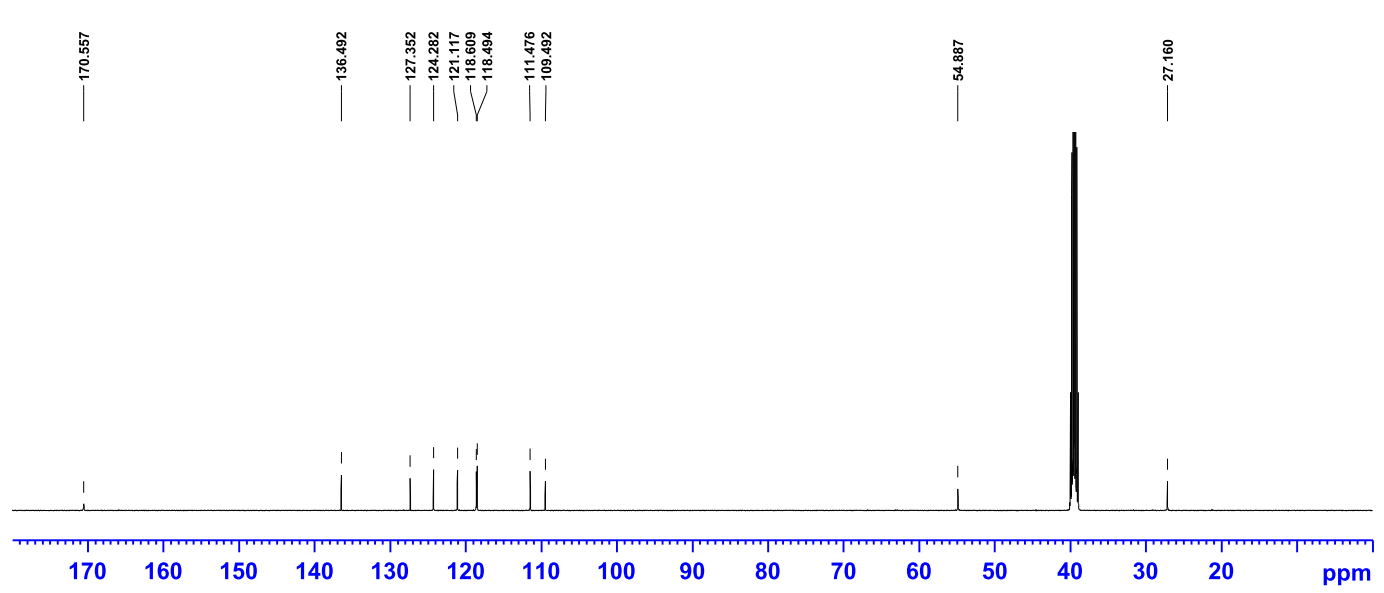


**(C)**


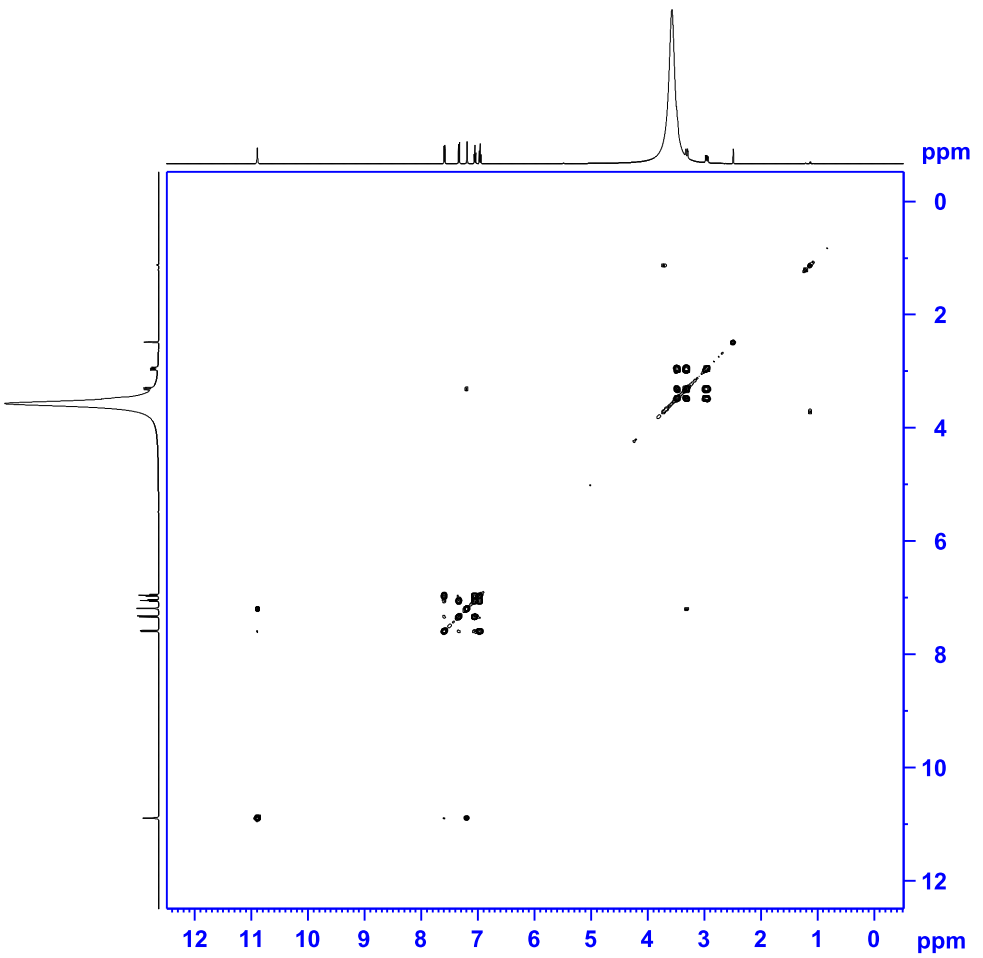


**(D)**


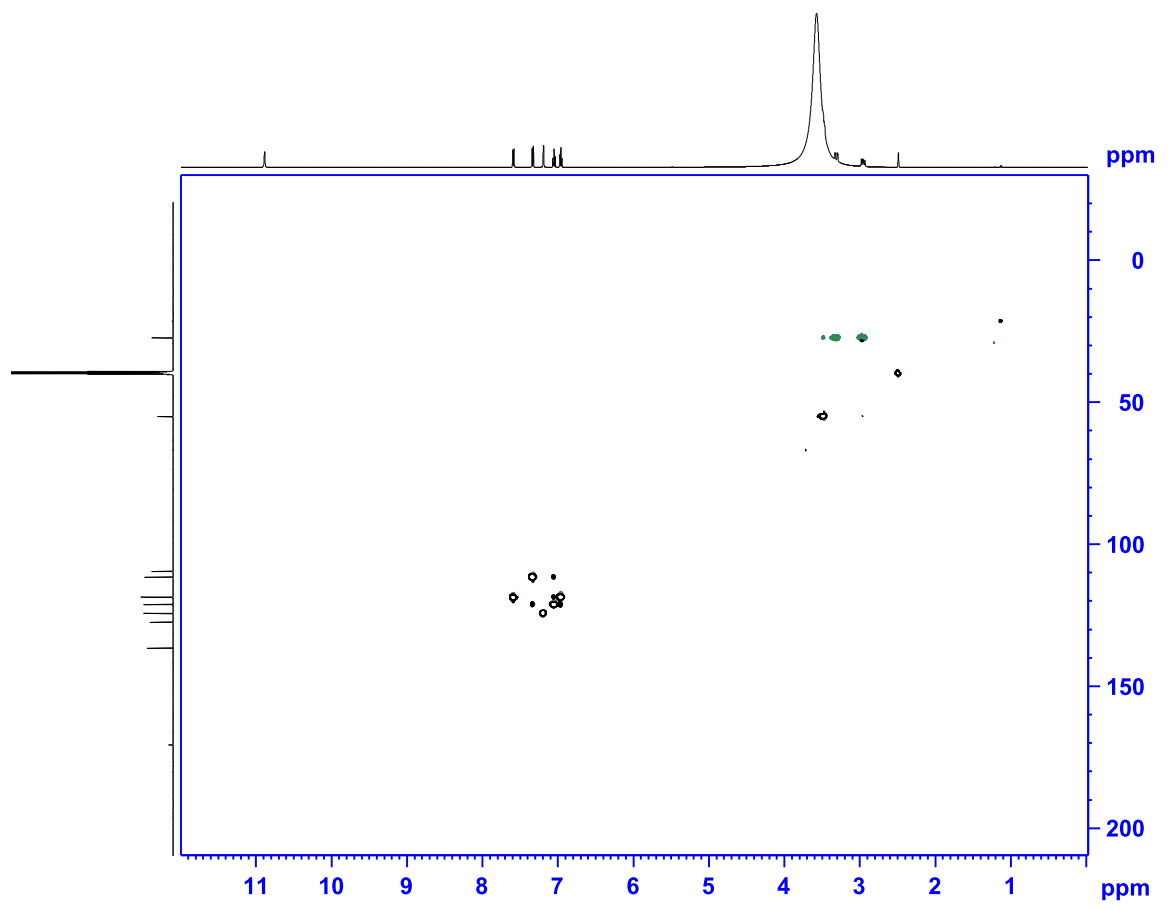


**(E)**


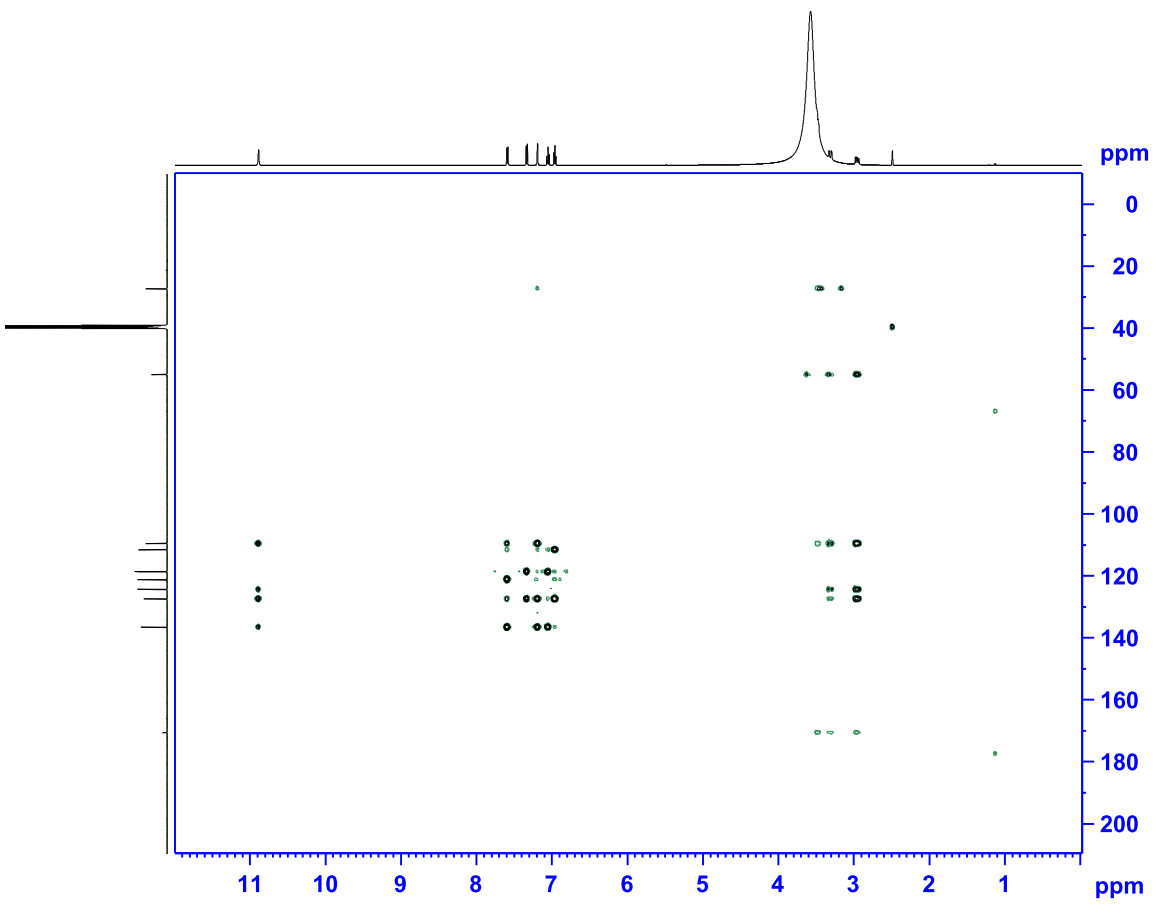


**Figure S5 (A)**


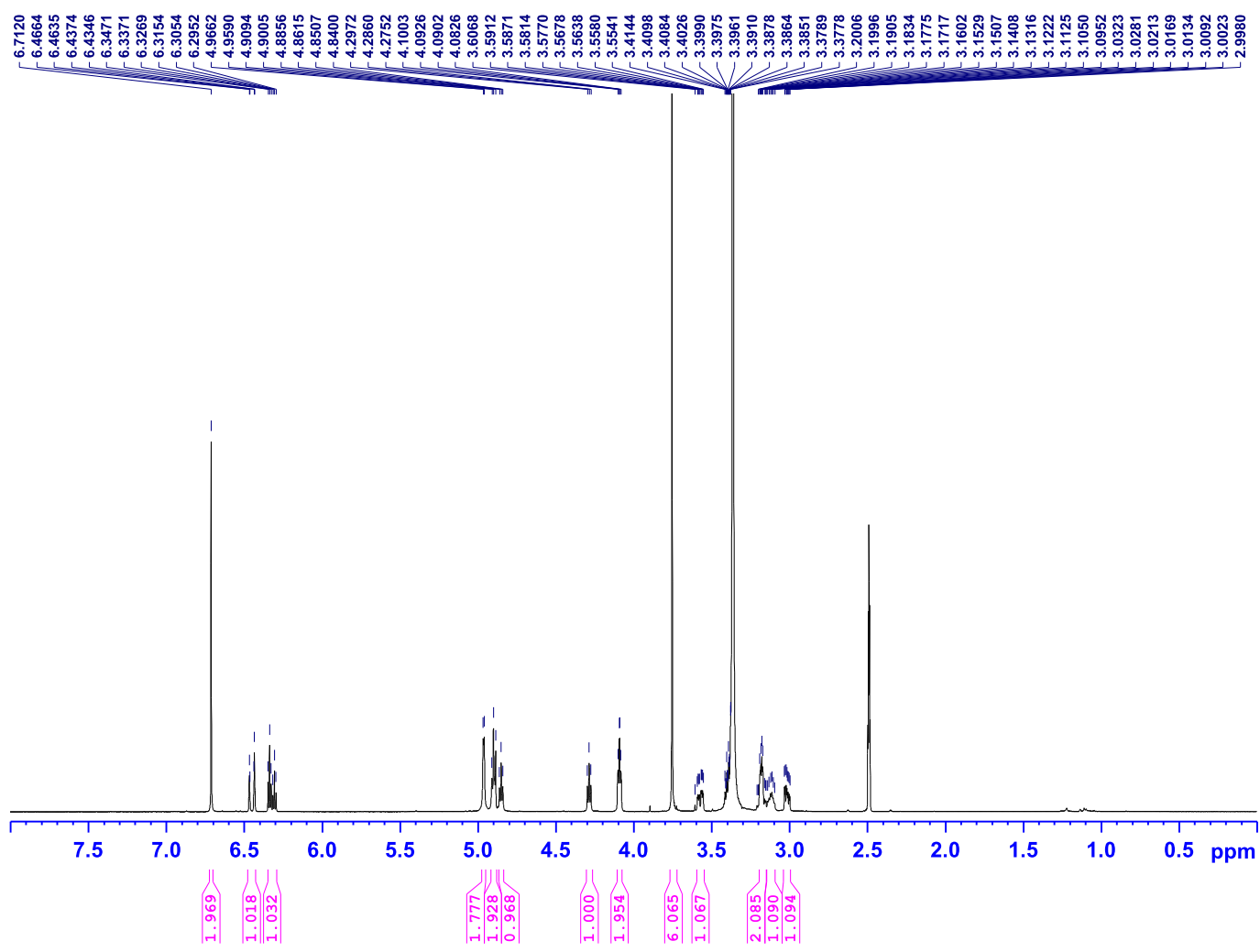


**(B)**

**
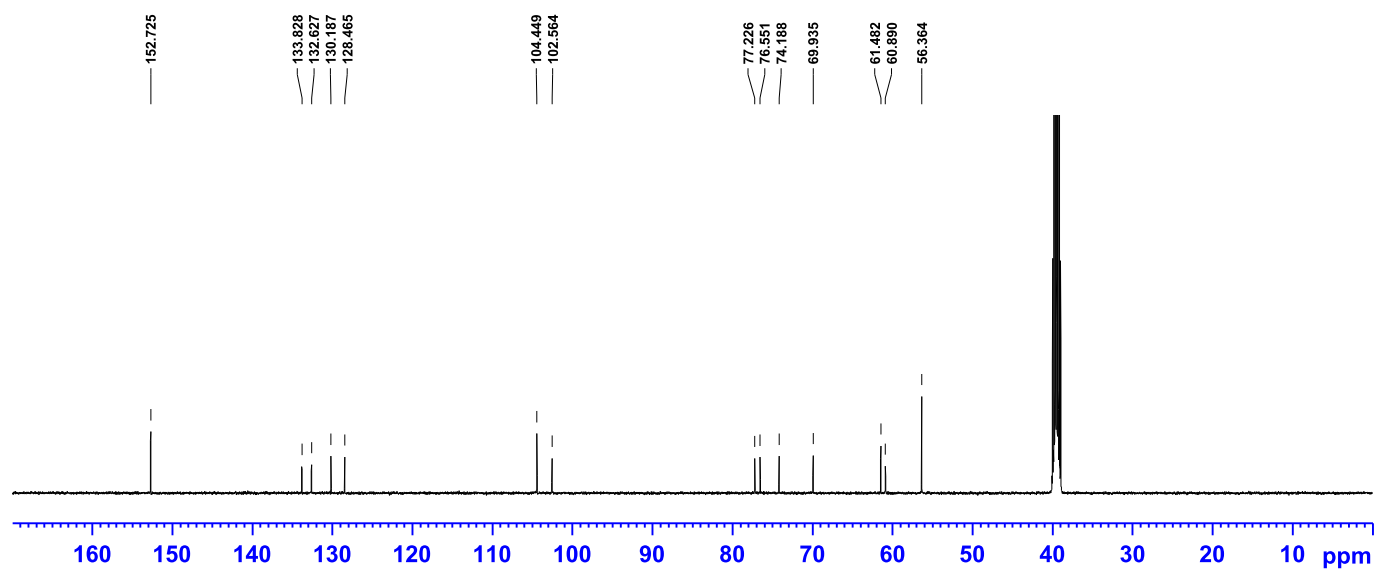
**

**(C)**


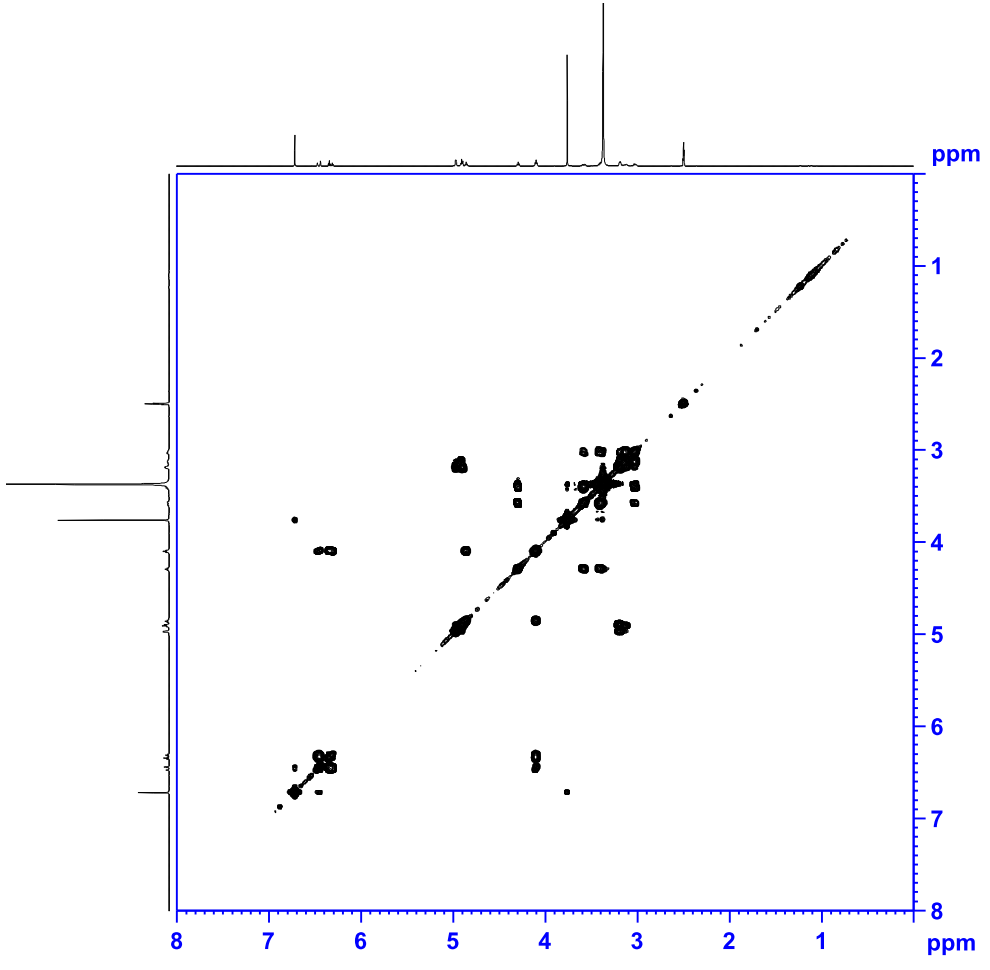


**(D)**


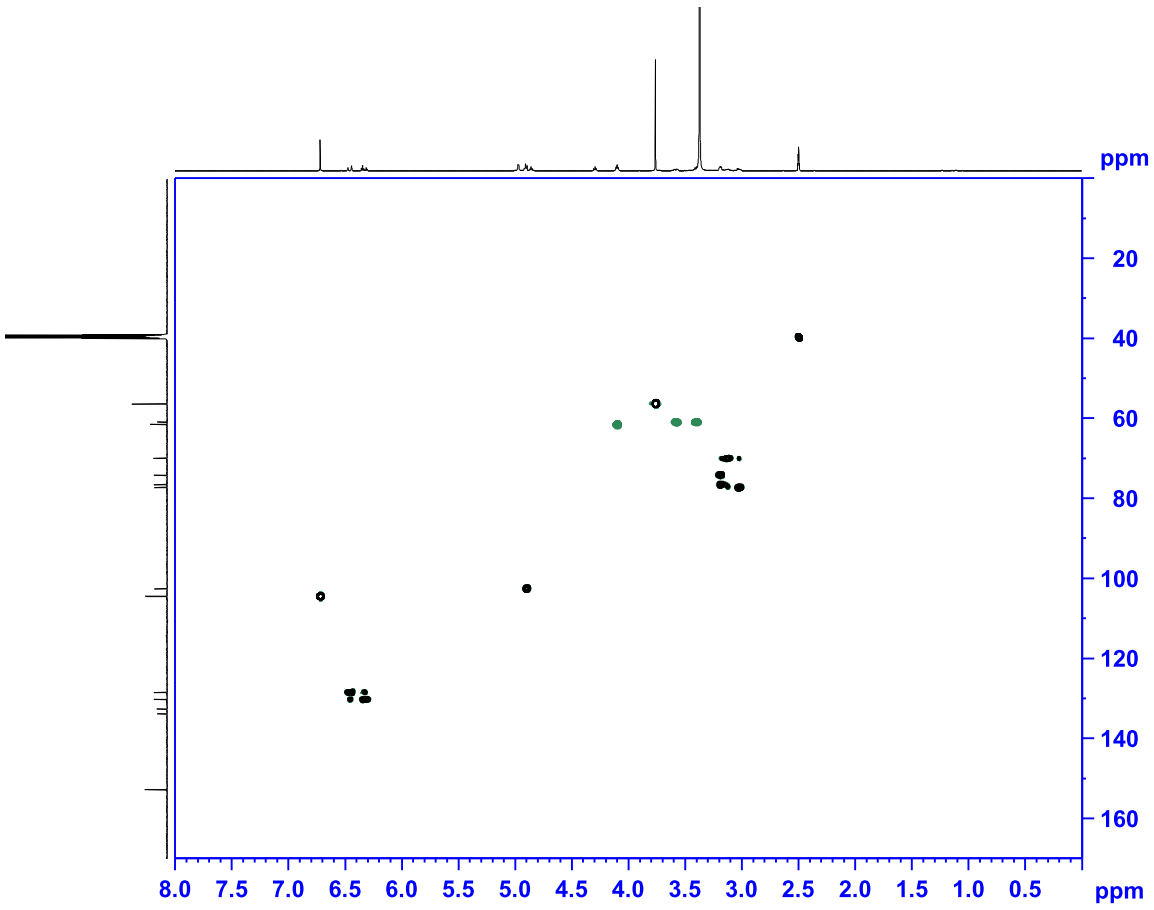


**(E)**


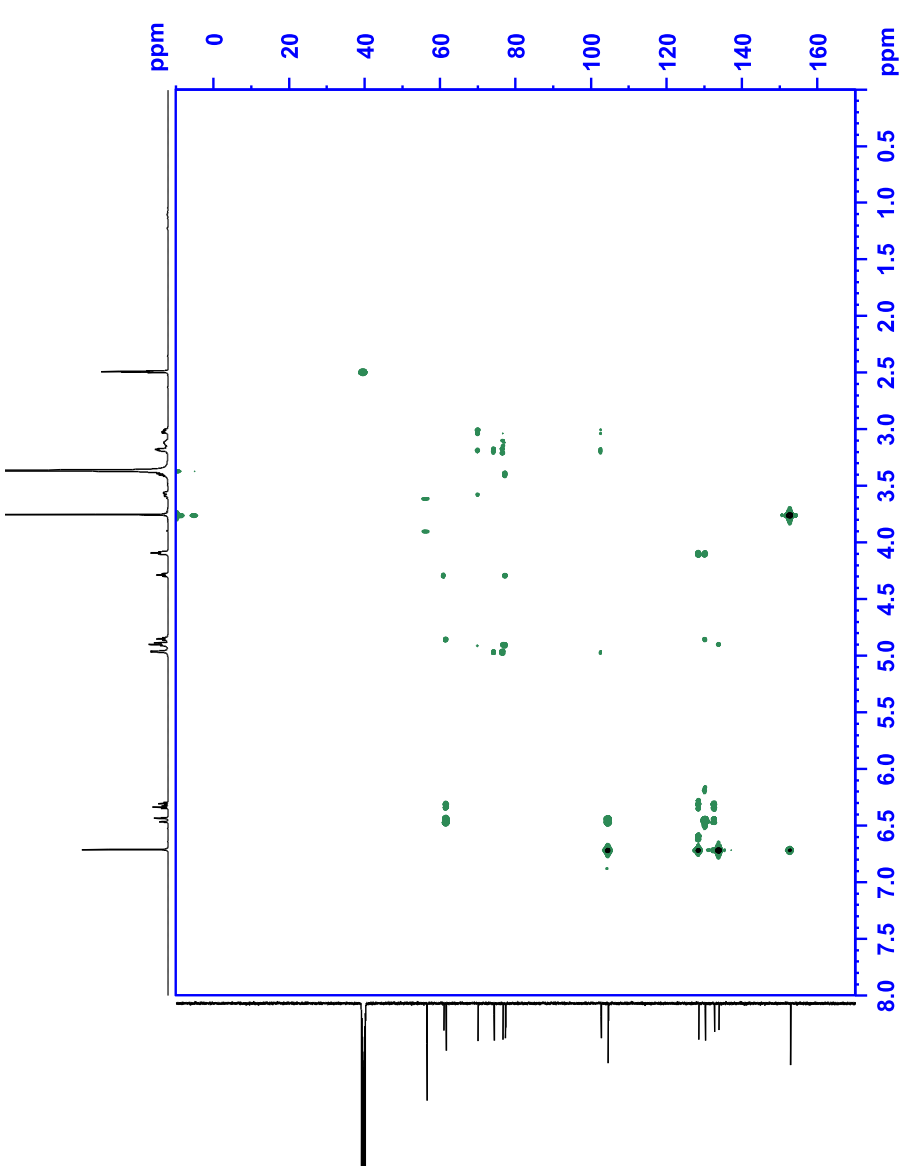

**Figure S6** Effects of **1** and **2** on the expression of estrogen receptor (ER)-β in human osteoblast cells. Cells were seeded in 96-well plates, and samples were added 24 h later. After five days, ER-β expression was detected with an ELISA kit according to the manufacturer’s instructions. Experiments were carried out in triplicate. * *p* < 0.05 and ** *p* < 0.01; data are presented as the mean ± standard deviation.
